# The Influence of Obturators on the Respiration of Patients with Maxillary Defects: A Clinical Study

**DOI:** 10.1371/journal.pone.0127597

**Published:** 2015-05-26

**Authors:** Xian Dong, Chenyuan Zhu, Yumei Qian, Fuqiang Zhang, Ting Jiao

**Affiliations:** 1 Department of Prosthodontics, Ninth People’s Hospital, affiliated to Shanghai Jiao Tong University School of Medicine, Shanghai Key Laboratory of Stomatology, 639 Zhi Zao Ju Road, Shanghai, PR China; 2 Department of Prosthodontics, Shanghai Institute of Health Sciences, 279 Zhou Zhu Gong Road, Shanghai, PR China; 3 Department of Prosthodontics, Branch Hospital of Shanghai Stomatological Disease Center, 1258 Middle Fuxing Road, Shanghai, PR China; The Ohio State University, UNITED STATES

## Abstract

The study evaluated the effects of obturators on respiratory function by analyzing the changes in nasal anatomic structures and physiologic function in maxillectomy patients with and without obturators. Twenty-six patients who underwent maxillectomy were chosen and rehabilitated with obturators by a single maxillofacial prosthodontist. The geometric shape of the nasal cavity, the nasal airway resistance, and the ratio of residual volume to total lung capacity (RV/TLC) were evaluated using acoustic rhinometry, rhinomanometry, and a pulmonary function test apparatus, respectively. All patients were tested twice, with and without their obturators. The results were statistically analyzed with a paired t-test. The nasal cavities (0–7 cm to the anterior nostril) of the patients with obturators had a significantly smaller volume ([-8.92, -0.60], *P* = 0.027), smaller effective nasal cross-sectional area MCA2 ([-3.80, -1,81], *P*<0.0001), increased airflow in the nasal cavity ([17.76, 147.39], *P* = 0.015), reduced nasal airway resistance ([-0.11, -0.02], *P* = 0.009), and reduced RV/TLC ([-5.32, -1.30], *P* = 0.004) compared with the patients without obturators. According to the results of this study, obturators can improve respiratory function by effectively decreasing the volume of enlarged nasal cavities as well as the nasal air resistance and volume of anatomical dead space after maxillectomy.

**Trial Registration:**

ChiCTR.org ChiCTR-PRNRC-14005136

## Introduction

Resection of the maxilla results in the disappearance of the maxillary sinus, turbinate defects, and communication between the mouth and the nasal cavity. These defects and communications may cause nasal airflow to change and ultimately influence a patient’s respiratory function. The airflow volume, airway resistance, and functional residual volume (which is also a part of respiratory function) may be affected by the resection of the maxilla. Previous quantitative simulation research indicates that these complications trigger obvious functional reductions in the filtration, warming and humidification of the air [1,2]. Obturators assist in restoring mastication, speech, and swallowing among maxillectomy patients. Kornblith et al. [3] conducted a clinical self-controlled experiment on the functional outcomes of the patients with and without obturators using the Obturator Functioning Scale (OFS) and discovered that obturators can dramatically improve patient quality of life. The resin base provides lip support, and the defective nasal cavity can recover to form an independent and relatively closed cavity [4,5]. Given our limited knowledge, however, little in vivo information is available regarding how and whether these form changes affect respiratory function. The purpose of this study was to evaluate the effects of obturators on respiratory function by analyzing changes in nasal anatomic structures and physiologic functions in the presence or absence of obturators in maxillectomy patients. The null hypothesis states that obturators do not influence the respiratory function of patients with maxillectomy defects.

## Materials and Methods

The protocol for this trial and supporting TREND checklist are available as supporting information; see S1 TREND Checklist and S1 Protocol.

This study was registered by the Chinese Clinical Trial Registry under registration number ChiCTR-PRNRC-14005136.This study was conducted in accordance with the Helsinki Declaration of 1975. The protocol (#2011–8) was approved by the Independent Ethics Committee of Shanghai Ninth People's Hospital affiliated to Shanghai Jiao Tong University, School of Medicine on the date of February 25, 2011. The authors confirm that all ongoing and related trials for this treatment intervention are registered.

### Participants

All patients seeking the rehabilitation treatment for unilateral maxillary defect at the Department of Prosthodontics, Shanghai Ninth People’s Hospital from March 2011 to March 2013 were recruited for this study. The following inclusion criteria were applied to determine whether the patients were eligible for this study: no history of chronic diseases of the upper respiratory tract, no acute infections of the upper respiratory tract in the previous one month, and no partial medication history of the nasal cavity. Female patients who were pregnant or menstruating were excluded. Any patients with an incomplete nasal septum was also excluded through clinical examinations and imaging studies. All of the patients who understood the study and were willing to participate signed the consent form and were recruited for the study over the following two years.

All patients received treatments for maxillary obturators by an experienced maxillofacial prosthodontist at our department. The measurements were performed at the time of the initial insertion of the obturator. Patients first performed the test without wearing the obturator. After the obturator was inserted into patient’s defect and the unified standard evaluation was settled, the same measurements were performed. The evaluation standards were as follows: 1) the obturator was well adapted to the defect 2) the retention was sufficient 3) the obturator had stable occlusion with the mandibular teeth 4) no obvious pain on mastication existed and 5) when the patient drank water while sitting upright, no fluid flowed from the patient’s nares.

### Nasal Airflow Measurements

#### Acoustic Rhinometry

Acoustic rhinometry was performed to measure the patients’ nasal cavity volumes and the two narrowest cross-sectional areas. An acoustic rhinometer (A1, Britain GM Equipment Corporation, UK) was used to evaluate the nasal geometry on both sides. The mean total volume from the nostril to 50 mm and 70 mm posterior, and the mean total volume of the nasal cavity (22–54 mm distal from the nostril) were measured. The mean minimal cross-sectional areas (MCA1, 0–22 mm from the nasal entrance; MCA2, 22–54 mm from the nasal entrance) were measured, according to previous studies [6].

#### Active Anterior Rhinomanometry

A rhinomanometer (NR6, Britain GM Equipment Corporation, UK) was used to measure the nasal airflow and resistance to airflow. The values were used to calculate the total nasal airway resistance (NAR). According to the recommendations of the International Committee on Standardization of Rhinomanometry [6,7], we obtained the flow measurements at P = 150 because at this pressure difference, there is a laminar airflow during inspiration. A trans-nasal reference pressure (150 Pa) was obtained from all participants before and after the obturator was placed in the mouth.

### The Functional Residual Capacity (FRC) Measurement

A pulmonary function test system (CHESTAC-8800-D, Japanese CHEST Corporation, Japan) was used to measure FRC. The gold standard re-breathing method (helium dilution method) recommended by the American Thoracic Society was used in the FRC examination[8,9].Using formulas, the testing system provides an accurate FRC value, as well as the residual volume (RV), total lung capacity (TLC) and RV/TLC based on the FRC. Patients who had histories of smoking did not complete this measurement.

### Statistical Approaches

This study was designed as a self-controlled experiment, and all of the measurements were performed by specialists at the clinic who were unaware of the objective of this study. SPSS (version 13.0, SPSS Corporation, U.S.) was used for statistical analysis, and all data were presented as the mean ± standard deviation (SD) to describe the data distribution. The one-sample Kolmogorov-Smirnov test was applied to the data distribution related to the obturator prosthesis placement (before or after), and the normal distribution data were analyzed with a paired *t*-test, while the non-normal distribution data were analyzed with the Wilcoxon signed-rank test. In this study, α = 0.05, β = 0.2,and 1-β = 0.8. The clinically significant level was when the power of the statistical test was larger than 0.8. To determine the sample size, prior to this study, we conducted preliminary experiments to calculate the sample size using the following equation [10]:

$$n={\left[\frac{(z_{\alpha }+z_{\beta })\sigma_{d}}{\delta }\right]}^{2}$$

where Z_0.05_ = 1.96 and Z_0.2_ = 0.842. σ_d_ means the standard deviation of the difference value of the matched group. δ means the average value of the difference of the matched group.

We calculated that the minimum sample size should be 25 patients.

## Results

### Patient information and participant flow

**Fig 1:**
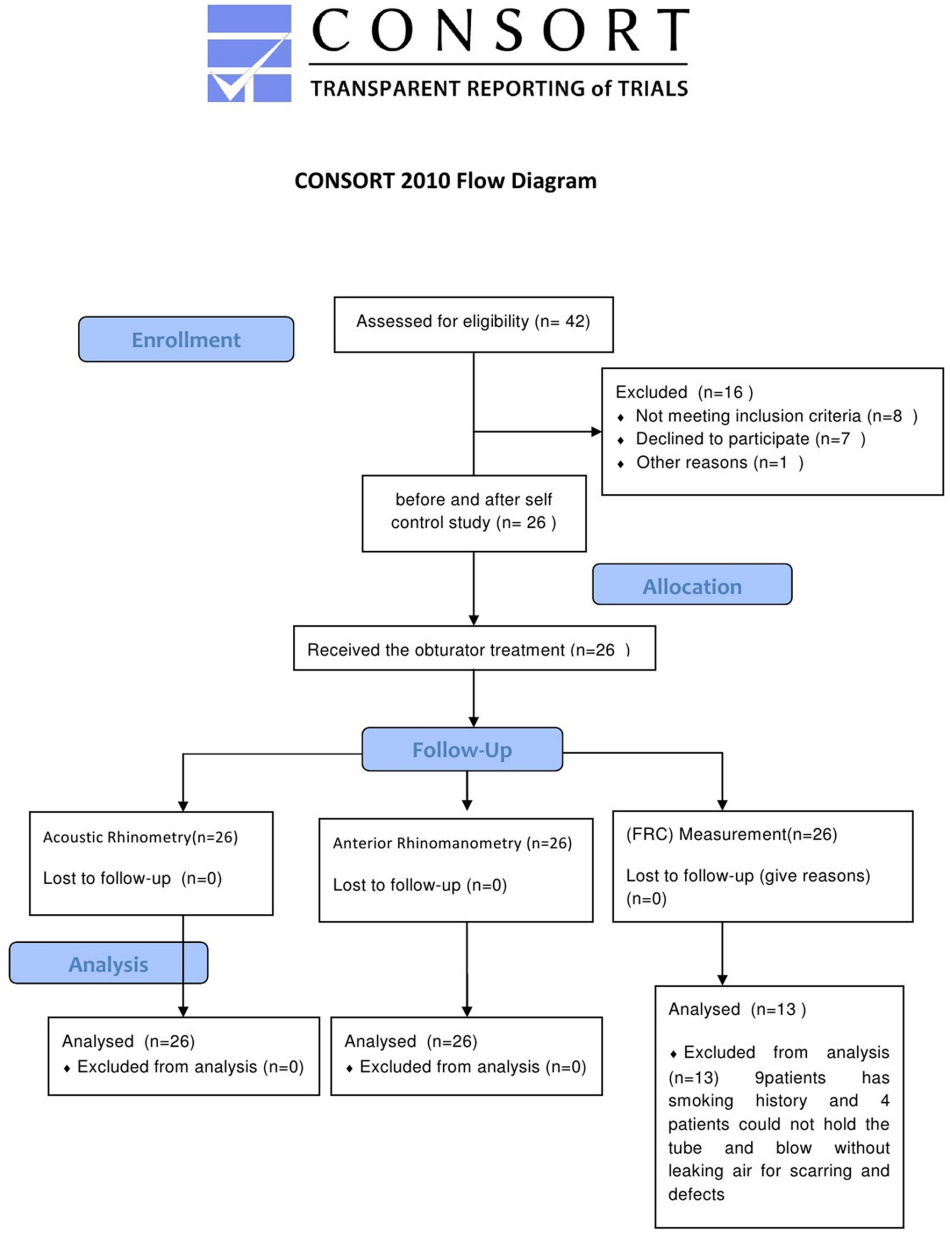
The completed CONSORT flowchart.

A total of 42 patients undergoing unilateral maxillectomy were recruited for this study, and 16 patients were excluded. The residual clinical data for the 26 patients (21–67, average age46.5) are presented in Table 1. The completed CONSORT flowchart of participants is shown in Fig 1. All of the 26 patients completed the obturator treatment and performed the nasal airflow measurements including acoustic rhinometry and active anterior rhinomanometry. The FRC test was performed on 17 non-smoking patients. Because of soft tissue cicatrice and defects, the lips of 4 patients were unable to hold the tube and blow without leaking air. The data of these 4 patients were excluded.

**Table 1:** Patient Information.

	All patients	
	NO	%
Gender		
Male	17	65.4
Female	9	34.6
Maxillectomy		
Total	17	65.4
Subtotal	9	34.6
Inferior nasal concha		
yes	2	7.7
no	22	84.6
n/a	2	7.7
Flap		
yes	13	50
no	13	50
Smoking history		
yes	9	34.6
no	17	65.4

### Geometrical Morphology Changes in the Nasal Cavity

The side of the nasal cavity that underwent maxillectomy was defined as the ill side, and the opposite side was defined as the healthy side. The anatomic structures of the two narrowest nasal cavity cross-sectional areas, MCA1 and MCA2, corresponded to the limen nasi and bottom of the inferior nasal concha, respectively. The mean total volume of the anterior nasal cavity (0–22 mm from the nasal entrance) was measured from the nostril to the limen nasi. The intranasal volume of the nasal cavity (22-54mm from the nasal entrance) stops at the lip of the inferior nasal concha and the nasal valve area. The mean total volume (0-70mm) indicates the anterior nasal cavity and the nasal proper cavity. Table 2 summarizes the patients’ testing results.

**Table 2:** The comparison of NV & NMCA before and after obturator placement (n = 26, $\bar{\text{x}}\pm \text{s}$).

		Vol0-5	Vol2-5	Vol0-7	MCA1	MCA2
		ml	ml	ml	cm^2^	cm^2^
Ill side	Before	16.53±11.79	15.03±11.53	23.39±16.16	0.53±0.20	4.34±2.52
	After	16.01±12.25	14.35±11.97	18.63±9.22	0.56±0.20	1.53±0.83
	P-Value	0.829	0.770	0.027*	0.396	0.000*
	95% CIs	-5.38,4.34	-5.46,4.09	-8.92,-0.60	-0.04,0.09	-3.80,-1.81
Healthy side	Before	8.64±3.97	6.93±3.62	19.72±16.64	0.65±0.28	1.63±0.85
	After	8.21±3.25	6.51±2.98	15.86±9.37	0.80±0.72	1.78±0.69
	P Value	0.320	0.271	0.063	0.156	0.333
	95% CIs	-1.31,0.44	-1.18,0.35	-7.95,0.23	-0.06,0.37	-0.16,0.46

Vol0-5: The mean total volume from the nostril to 50 mm, Vol0-7: The mean total volume from the nostril to 70 mm posterior, Vol2-5: The mean total volume of the nasal cavity (22–54 mm distal from the nostril). MCA1: The mean minimal cross-sectional areas (0–22 mm from the nasal entrance), MCA2: The mean minimal cross-sectional areas 22–54 mm from the nasal entrance.

*means comparing the obturator placement before and after; P<0.05.

**Fig 2:**
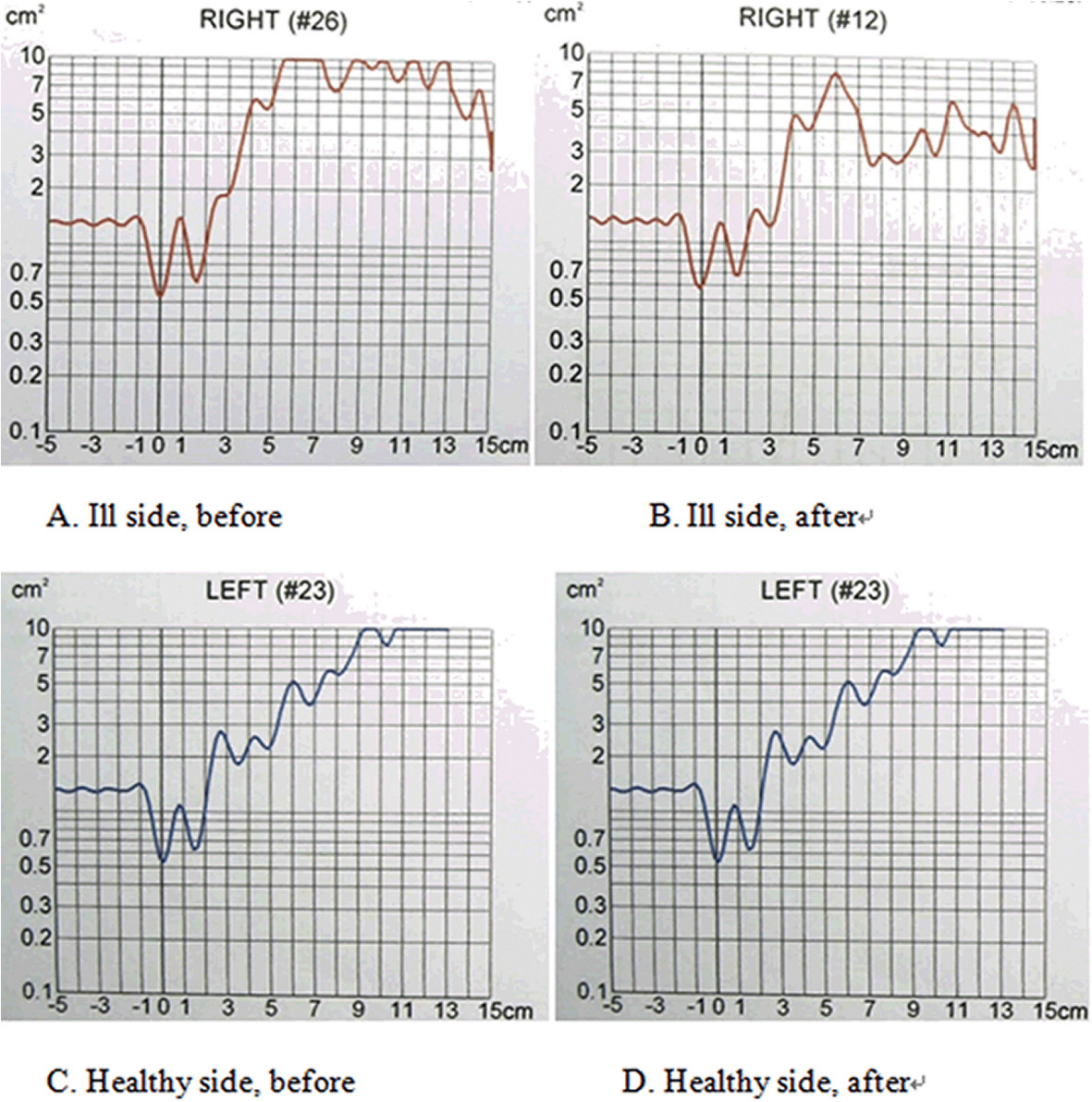
The nasal cross-sectional area versus the distance curves of a patient before and after obturator placement. A-D shows the significant geometrical morphology and volume changes of the cavity after obturator placement on the ill side. The before and after curves of the healthy side are similar, with minimal changes.

As shown in Table 2, the nasal volumes (NV) and minimum nasal cross-sectional areas (MNCA) of the side with the defect were greater than those of the healthy side before the obturator was inserted. After the obturator was inserted, the NVs decreased, particularly section Vol 0–7, which showed a significant reduction (P = 0.027, power = 0.61). For the NMCA, the MCA1 and MCA2 of the healthy side and the MCA1 of the ill side did not show a significant change after the obturator was inserted. However, the MCA2 of the ill side showed a significant decrease after insertion (P<0.0001, power = 0.99). Fig 2 show the nasal cross-sectional area versus distance curves of a patient before and after obturator placement.

### Airflow Changes in the Nasal Cavity

The airflow quantity and nasal airway resistance under the internal differential pressure of 75 Pa/150 Pa in the nasal cavity was obtained by measuring the nasal airway resistance. Table 3 provide the testing results.

**Table 3:** The airflow of the nasal cavity and nasal air resistance (NAR) at the differential pressure 75/150 Pa before and after obturator placement (n = 26, ˉ x ±s).

		Airflow of the nasal cavity		Nasal air resistance(NAR)	
		75 Pa(ml/s)	150Pa (ml/s)	75 Pa(ml/s)	150Pa(ml/s)
Ill side	Before	193.54±129.48	263.88±174.09	0.92±1.22	1.73±1.97
	After	179.88±98.18	260.38±138.04	0.52±0.34	0.72±0.49
	P_Value	0.542	0.586	0.077	0.007*
	95% CIs	-59.19, 31.88	-60.83, 53.83	-0.85,0.05	-1.72, -0.29
Healthy side	Before	249.73±115.30	350.77±149.66	0.42±0.29	0.61±0.41
	After	256.92±114.94	370.27±156.75	0.41±0.33	0.56±0.41
	P_Value	0.901	0.291	0.925	0.302
	95% CIs	-19.63,34.02	-17.74,56.74	-0.09,0.08	-0.17,0.05
Sum	Before	443.27±181.87	614.65±223.03	0.25±0.21	0.36±0.25
	After	436.81±147.32	630.65±221.29	0.21±0.14	0.27±0.15
	P_Value	0.754	0.015*	0.057	0.009*
	95% CIs	-54.61,40.07	17.76,147.39	-0.27,0.00	-0.11,-0.02

*means comparing the obturator placement before and after; P<0.05.

As shown in Table 3, regardless of the differential pressure, the airflow of the nasal cavity’s ill side decreased after obturator insertion, while the airflow of the healthy side increased. The sum of the nasal cavity airflow after the obturator insertion also increased under the differential pressure of 150 Pa (P = 0.015, power = 0.712). However, the nasal airway resistance decreased after the obturator placement, and when under the differential pressure of 150 Pa the resistance changes of the ill side and the whole nasal cavity were significantly different (P = 0.009, power = 0.801).

**Fig 3:**
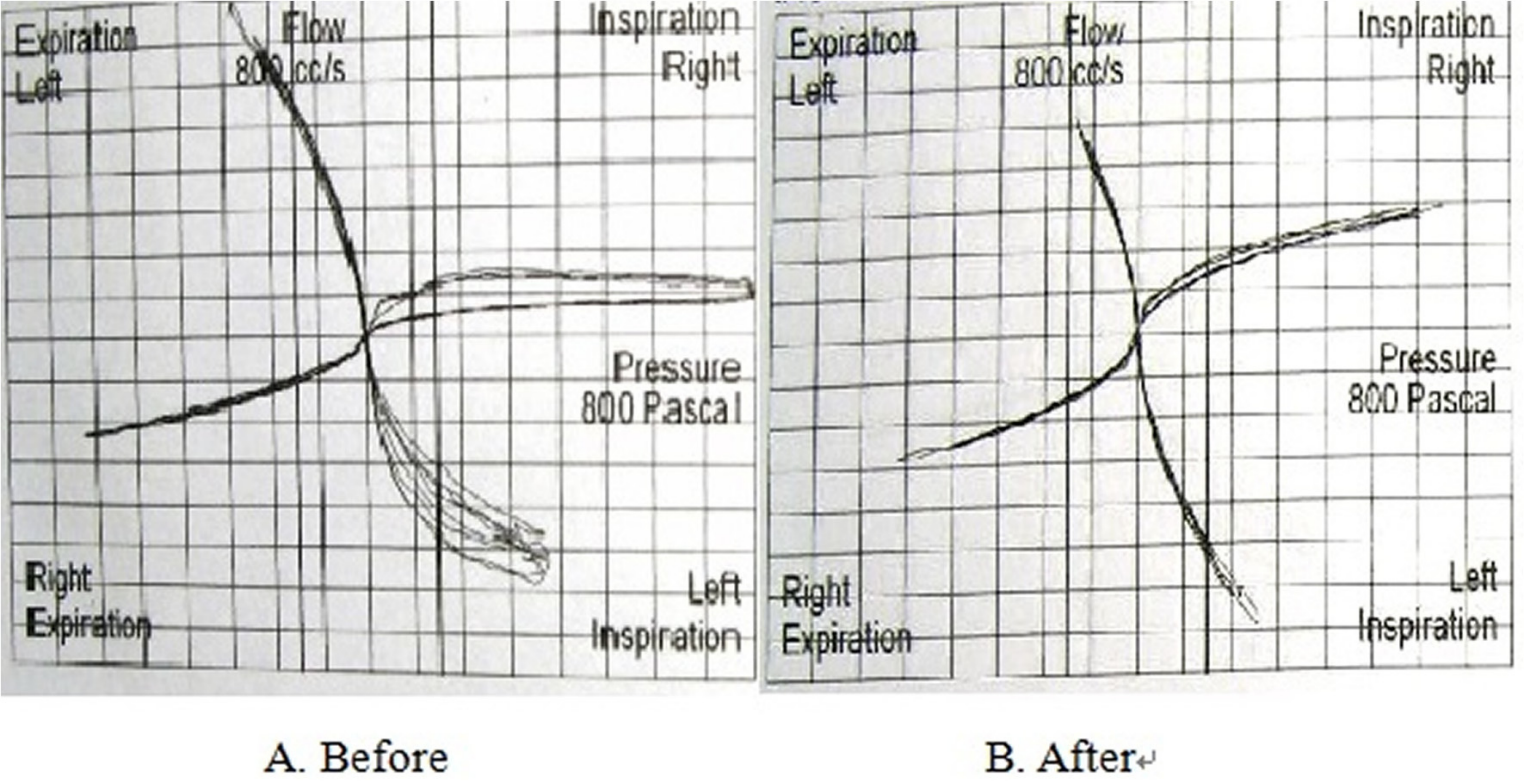
The nasal cavity pressure-flow curves of a patient before and after obturator placement (the right side contains the defect). A. Before obturator placement, the airflow of the ill side was significantly smaller than that of the healthy side; during inspiration, airflow was not steady or consistent. B. After obturator placement, the airflow on both sides increased during inspiration and expiration.

Fig 3 show the nasal cavity pressure-flow curves of a patient before and after obturator placement (the right side is the defect side).

### Residual Volume Changes in the Upper Respiratory Tract

After wearing the obturator, the RV/TLC significantly decreased by 7.9% (P = 0.004, power = 0.932). Refer to Table 4 for the results.

**Table 4:** The RV/TLC of patients before and after the obturator placement.

	％(x±s)	95%CIs	P_value	Power
Before	40.51±9.81	-5.32, -1.30	0.004*	0.932
After	36.74±8.40*			

*means comparing the obturator placement before and after; P < 0.05.

## Discussion

This study evaluated the influence of obturators on the respiration of patients with maxillary defects using a self-controlled experiment. The null hypothesis was rejected, and the results demonstrated that the obturator invention decreased the nasal surface and volume of the defect, decreased the nasal airway resistance, and increased the sum of the nasal cavity airflow. In addition, the obturator insertion separated the mouth from the nasal cavity and decreased the volume of the dead space, thereby prohibiting the RV/TCL to return to normal.

### Obturator Influence on the Geometric Morphology of the Nasal Cavity

Based on the study results, after maxillectomy, the volume of the patient’s ill side showed a significant increase compared to the healthy side. The section Vol 2–5 of the nasal valve area showed the largest volume change (116.88%), and MCA2 expanded to 166.26% compared to the size of the healthy side. When the obturator was inserted, the Vol 0–7 and MCA2 data decreased significantly and were approximately the same size as the healthy side. These results were comparable to those obtained from computational numeric simulation by Qian et al[2,11]. The authors reported that the surface area and volume of the defect of a patient’s nasal cavity increased postoperatively. However, the statistical power of Vol0-7 didn’t reach the clinical significant level; the results of MCA2 might be more reliable.

MCA2 was measured at the lip of the inferior nasal concha and the nasal valve area. On the chart, the MCA2 area equaled the cross-sectional area when the distance was within a 2–5 cm section. Maxillectomy is usually accompanied by a partial or total excision of the inferior nasal concha, leading to a communication between the nose and mouth, which changes the geometric morphology of the patient’s nasal cavity. The nasal valve area is the narrowest section of the nasal cavity and is greatly influenced by the form of the inferior nasal concha. After surgery, significant damage of the nasal valve area was observed. When the obturator was inserted, it became the bottom of the nasal cavity, thereby closing the cavity and causing its volume to decrease, so that the narrow section of the nasal valve area was better restored. Clinically, these changes, which resulted from inserting the obturator, helped to normalize the patient by positively influencing the nasal airflow and helping it to sufficiently touch the nasal cavity.

Note that there was no statistically significant change in the MCA1 area before and after obturator placement (0.53±0.20 cm², 0.56±0.20 cm²). Some scholars have obtained a normal nasal cavity volume of 0.57±0.08 cm² [12], which align with this experimental result and indicate that inserting an obturator does not change the limen nasi structure. MCA1 was the cross-sectional area of the limen nasi when the distance was 0 cm in the curve chart. The limen nasi is the entrance for the air entering the nasal cavity. Because the airway suddenly narrows in this area, the limen nasi significantly influences the nasal airway resistance. Therefore, neither the maxillectomy nor obturator should touch this structure.

### Obturator Influence on Nasal Airway Resistance

Nasal airway resistance is primarily caused by the limen nasi and is also influenced by the hyperemia status of the turbinates [13,14]. In normal situations, the hyperemia status of the inferior nasal concha appears alternately on each side during a period of 1–7 hours, which is known as the physiological turbinate cycle or the nasal cycle. Therefore, the standard deviation of airflow was relatively high, both before and after inserting the obturator. However, the nasal cycle does not change the total nasal airway resistance [15]. In this experiment, the total nasal airway resistance results were more reliable and significant than those of the ill or healthy sides. The nasal airway resistance measurement is influenced by many factors [16], including the patient’s position, environmental elements (temperature, humidity, noise etc.), medications (e.g., nasal angiotonics, aspirin, etc.), movements, emotional changes, and pregnancy. Considering the factors mentioned above, this experiment was designed to decrease the influence of those factors on the nasal airway resistance as much as possible.

Regarding the sum of the right and left nasal cavities, the airflow increased, and the nasal airway resistance decreased after the obturator placement, particularly under the 150 Pa differential pressure. These results were consistent with the numeric simulation results by computational fluid dynamics [11,17]. The authors in that study reported that the anatomically invalid cavity and the resistance to airflow reduced after the restoration of a maxillary defect with a prosthesis. However, the statistical power of the airflow (150Pa) didn’t reach the clinical significant level; the results of nasal air resistance might be more reliable.

Additionally, the normal value of the nasal airway resistance depends on different studies and is related to the age, gender and nasal cavity anatomy. According to measurements taken by Bu Guadiana from 1145 Chinese citizens, the nasal airway resistance for adults was 0.126–0.328 kPa/L•s [15]. Another study concluded that normal nasal airway resistance should measure below 0.294 kPa/L•s: a value between 0.294 and 0.686 kPa/L•s suggests the existence of a moderate blockage, and a value above 0.686 kPa/L•s suggests a serious blockage [18]. It also concluded that if the nasal airway resistance measured above 0.294 kPa/L•s, then the patient would sense the nasal airway blockage. Based on these values, the patients who underwent maxillectomy in this study displayed moderate resistance, and the obturator helped the nasal airway resistance close to a normal level.

Measuring nasal airway resistance provides an objective method of evaluating nasal ventilation. However, the objective measurement of ventilation does not always correlate with the subjective feeling of obstruction [19,20,21]. In this experiment, patients were told to press on one side of the nares and report the sensation of obstruction on the other side. Some patients felt obstructed before obturator placement and felt relieved after the placement, while others felt the opposite sensation. Some patients did not feel obstructed before or after obturator placement. These subjective feelings did not always align with the patients' AR and NAR values. Clinically, the patient places more emphasis on subjective feelings. However, from the doctor's perspective, both the improvement of nasal ventilation and the relief of the patient's subjective symptoms should be considered.

### Obturator Influence on Nasal Conditions

The values from the postoperative and obturator placement measurements were used to calculate the relationship of nasal airflow and nasal geometry with nasal air conditioning. Papp et al. [7] concluded that high nasal volumes and high nasal airflow showed a significant and positive correlation with higher temperature gradients and a negative correlation with water gradients. Patients commonly complain of nasal drying, crusting, and secretions accumulating after unilateral maxillectomy but feel better after wearing the obturator. The results of this study may explain these findings. The ability to maintain moisture in the nasal cavity is dependent on an intact sinonasal mucosa. We hypothesized that if patients lost the inferior turbinate during surgery, the over-enlargement of the nasal airways and the altered nasal resistance in the wider nasal airways might reduce the contact of the inspired air with the surrounding nasal respiratory mucosa. In addition, the over-enlarged volume may cause over heating in the nasal mucosa and result in wasted energy, leading to feelings of discomfort. Obturators reduced the over-enlarged volume of the nasal cavity, reduced nasal resistance leading to closer contact of the inhaled air with the sinonasal mucosa and subsequently causing a relatively normal exchange of heat and water.

### Obturator Influence on Residual Airway Volume

The existence of nasal cavity resistance helps to create the negative pressure in the chest during inspiration, which expands the pulmonary alveoli and enlarges the area for gas exchange. This resistance also extends the amount of time that gas stays in the pulmonary alveoli during expiration [13,14]

Because patients with a maxillary defect experienced a change in the structure of the nasal cavity on the ill side, they also showed increases in the anatomical dead space of the upper respiratory tract and nasal resistance. Their breathing airflow also changed. To determine whether these changes would influence the respiratory oxygen and blood exchange volume, this experiment further recruited 17 patients with no smoking history and conducted a pulmonary ventilation function test, which was also preliminary research on the RV/TCL value of the obturator before and after placement. In clinical medicine, the RV/TCL is an important measure used to diagnose emphysema or other diseases. In healthy people, the RV/TCL values should be <35% [22]. The results of this experiment showed that the RV/TCL values were >35%, with an average of 40.42% before obturator placement. Therefore, the maxillary defect might cause gas retention in the respiratory tract, which is a type of obstructive ventilation disorder that may result in decreased alveolar ventilation volume. According to the mathematic formula for the definition of alveolar ventilation [22]:

$${\text{V}}_{\text{A}}＝({\text{V}}_{\text{T}}-{\text{V}}_{\text{D}})＊\text{BR}$$

V_A_: Alveolar ventilation, V_T_: Tidal Volume, V_D_: Dead Space volume (includes both the anatomic dead space and the physiologic dead space)

, BR: breathing rate

Note that when no obvious change in the patient's tidal volume or breathing rate exists, a larger dead space will result in a smaller alveolar ventilation volume. The alveolar ventilation volume directly indicates the air and blood exchange volume. Thus, it can be deduced that the alveolar ventilation volume of patients with maxillary defects may be lower than the normal value. However, after obturator placement, the RV/TCL values all decreased, and only two slightly increased, but the increase was <1%. After placement, the RV/TCL values of 5 patients decreased to a normal level, and all 13 values decreased to an average of 37.45%, which approximated the maximum normal value of 35%. Therefore, the obturator insertion separated the mouth from the nasal cavity and decreased the volume of the dead space. We deduced that the alveolar ventilation volume increased after the obturator insertion and that the gas volume in the oxygen and blood exchange also increased.

Qian and Gai [8,13] observed the generation of spacious low velocity vortices throughout the entire maxillary defect during respiration after surgery. They found that after obturator placement, vortices reduced significantly and only slightly existed at the top turbinate, which is close to the healthy side. The vortices were thought to be the source of the wasted energy, thereby preventing the mixing of air within the center of the air stream and impacting pronunciation. Obturators can reduce the energy waste, help the respiration, and protect the exposed mucous membrane.

Therefore we hypothesize that the alveolar ventilation volume of patients with maxillary defects may be lower than the normal value. If the alveolar ventilation is chronically lower than normal, it will likely cause alveolar expansion and increase the risk of emphysema and other diseases. However, the obturator decreased the RV/TCL values, thus decreasing the anatomical dead space while increasing the alveolar ventilation volume. This process improved the patient's respiratory function. Clinically, when fabricating an obturator, maxillofacial prosthodontists and physicians should also consider the influence that the maxillary defect has on respiratory function, and they regularly perform follow-up examinations of these patient's respiratory functions.

### Limitations

Respiratory function is influenced by many factors and can be evaluated using many techniques [23,24,25]. This experiment only chose the most direct measures related to obturator placement, and the observation period was limited to the moment of initial obturator delivery. In addition, only 26 patients could be recruited during the two years. As a result, we were not able to conduct a multivariable analysis such as a mixed-effects model to analyze the factors of gender, method of maxillectomy, flap, baseline condition and so on. A more comprehensive result should be obtained from a long-term research study with a larger sample size and a multivariate data analysis. Furthermore, most patients came to our department after surgery with their defects. Therefore, we cannot obtain baseline (preoperational) values or compare another experimental group with another type of reconstruction (e.g., vascular free flap). Future studies should be conducted in these areas.

## Conclusions

Obturators can decrease nasal cavity volume and nasal airway resistance, thereby increasing the airflow passing through the nasal cavity. These changes are helpful for improving the nasal response to heat and humidity. Obturators also increase the alveolar ventilation volume, which might increase the oxygen exchange into blood and improve respiratory functions. Therefore, it is necessary to rehabilitate the maxillary defect using obturator prostheses as soon as possible after surgery.

## Supporting Information

S1 Protocol English version of protocol. (PDF)

S2 Protocol Original version of protocol in Chinese including backgrounds, objectives, research content and research procedures. (PDF)

S1 TREND Checklist The TREND statement checklist. (PDF)
