# Supplementary material for: The Influence of Obturators on the Respiration of Patients with Maxillary Defects: A Clinical Study
Source: PLoS One. 2015 May 26;10(5):e0127597. doi: 10.1371/journal.pone.0127597 (PMC4444186; doi:10.1371/journal.pone.0127597)
Supplement: S1 Protocol — (PDF) [file pone.0127597.s001.pdf]

Research protocol

1. Backgrounds

Maxillary defects, with the highest incidence of facial defects-58.8%, are the most common oral and maxillofacial defects in China (1). They are mainly caused by tumor resection. These patients suffer the hypernasal speech, fluid leakage in their nasal cavities, impaired masticatory function, and, some of them have cosmetic deformities in various degrees after surgery. Although some scholars believe that the surgical reconstruction is better than prosthesis in restoring speech(9), but artifical materials prosthesis rehabilitation for patients according to varied anatomical and histological structure of maxillofacial region is more preferable to surgery when defect is large , blood supply is poor and patient is too old to withstand several surgeries. Maxillofacial prostheses technology has become one of the signs of stomatology technology in developed countries (10,11). Obturators are used to rehabilitate their hypernasal speech, leakage and impaired mastication, highly improving the quality of their lives postoperatively(2-5). However, some patients still have symptoms like respiratory congestion, tinnitus or not clear enough to pronounce after repair. Why there are a series same clinical symptoms on these patients? Why do not all patients appear the above aymptoms after obturator treatment?

Currently,little research studied the influence on breathing and pronunciation change mechanism of patients with maxillary defects and existing theory can not make a clear interpretation of these clinical symptoms and problems.Resection of the maxilla results in the disappearance of the maxillary sinus, turbinate defects, and communication between the mouth and the nasal cavity. These defects and communications may cause nasal airflow to change and ultimately influence a patient’s respiratory function. The airflow volume, airway resistance, and functional residual volume (which is also a part of respiratory function) may be affected by the resection of the maxilla. Some numerical simulation researches have indicated these complications may trigger an obvious functional reduction in the filtration, warming and humidification of the air(6).An obturator assists in restoring mastication, speech, and swallowing in maxillectomy patients. Lip support is provided by the resin base, and the defective nasal cavity can recover to form an independent and relatively closed cavity(1,7). However, Given our limited knowledge, little information can be found as to how and whether these form changes affect respiratory function. T

Nasal dryness and crusting are common complaints from patients with maxillectomy defects. These symptoms are attributed to changes of upper airway structures and airflow dynamics(7). However, there are few studies on postoperative airflow dynamics patterns. Research in this area revealed that computational fluid dynamics (CFD) can be a useful tool for evaluating nasal airflow patterns during respiration(8).

At present, the rehabilitation of maxillary defects mainly adopts a hollow obturator. According to the obturator top wall is retained or not ,the obturators can be divided into closed and open type. Studies have shown that open type hollow obturator prosthesis can reduce 6.55% to 33.06% of the weight(12), thus minimize loading for abutment teeth and contribute to retention and stability.Meanwhile ,obturators can also be divided into high,medium and low according to their buccal extension.However,the optimal height and shape of obturator remains unknown.Yang Liu et al.advocated the use of high obturator; Aramany indicated that low obturator is more

conducive to unobstructed breathing and improves speech intelligibility; Drane believes that the nose shorter extension helps to improve speech with large defect, however,height of obturator appears no relation with speech with relatively small defects.but if the defect area is small, blocker extension length on the pronunciation is a little effect. The lack of adequate theoretical guidance for the height and shape of(13-15)blocking device selection.Up to now,theory regarding to the optimal height and shape of obturator is insufficient and needs deeper research.

In recent years, the rise of biomedical and engineering , some research methods in engineering is rapidly introduced into medical field. Steady flow of human respiratory can be simulate be simulated using CFD.In previous study, the analysis of airflow characteristics in airway of a patient with a closed prosthesis after unilateral maxillectomy explained preliminarily the influence of obturator on airflow pattern in the defected cavity.However,comparison between inspiration and expiration was not made and no suggestion was put forward as to the optimal height of obturator from the perspective of hydrodynamics.

The purposes of this study were (1) to evaluate the effects of obturators on respiratory function by analyzing changes in nasal anatomic structures and physiologic functions in the presence or absence of obturators in maxillectomy patients, (2) to analyze the influence of postoperative upper airway structures on airflow patterns by means of numeric simulation of the airway using CFD on a three-dimensional(3D) reconstructed upper airway model, (3) to reconstructed an airway model wearing different prosthesis and analyzed the steady flow during both the the inspiratory and expiratory phases,in the hope of explaining the change of flow in the defect side and the potential effect of such change,thus providing reference for designation of obturators, (4) Make an understanding of sound transmission caused by variation of airflow in the altered vocal resonance cavity through fluid and acoustic simulation in order to discover the factors which caused sound change and verify that sound is improved after maxillary rehabilitation.

Refrences:

1. Zhao Y (2004) Maxillofacial Prosthetics:Outline:(pp.2~6). Xi'an: World Publishing Corporation. .

2. Ortegon SM, Martin JW, Lewin JS.A hollow delayed surgical obturator for a bilateral subtotal maxillectomy patient: a clinical report [J]. J Prosthet Dent, 2008,99(1):14-18.

3. Barnouti L. Caminer D.Maxillary tumours and bilateral reconstruction of the maxilla [J].ANZ J Surg, 2006,76(4):267-269.

4. Xing GF, Jiao T, Sun J, Jiang YL. Evaluation of the speech outcomes in patients with unilateral maxillary defect rehabilitated with maxillary obturator prosthesis. [J]. Shanghai Kou Qiang Yi Xue. 2005 Aug;14(4):352-4.

5. Itsuki Murase.In-vivo modal analysis of maxillary dentition in a maxillectomy patient wearing buccal flange obturator prostheses with different bulb height designs[J]. Nihon Hotetsu Shika Gakkai Zasshi, 2008, 52(2):150-159.

6. Ozlugedik S, Nakiboglu G, Sert C, Elhan A, Tonuk E, et al. (2008) Numerical study of the aerodynamic effects of septoplasty and partial lateral turbinectomy. The Laryngoscope 118: 330-334.

7. Beumer J III MM, Esposito SJ (2011) Esposito SJ. Maxillofacial Rehabilitation: Surgical and Prosthodontic Management of Cancer-Related. Acquired , and Congenital Defect of the Head and Neck,ed3. . Chicago:Quintessence: 155-212.

8. Ishikawa S, Nakayama T, Watanabe M, Matsuzawa T.Visualization of flow resistance in physiological nasal respiration:Analysis of velocity and vorticities using numerical simulation.Arch Otolaryngol Head Neck Surg 2006;132:1203–1209.
9. Weng Yanqiu,Sun Jian,Chen Yang,et al.Evaluation of speech function after prosthesis rehabilitation or reconstruction surgery for maxillary defects,2005,3(1):43-47.
10. Ducic Y. An effective, inexpensive, temporary surgicalobturator Following maxillectomy[J].Laryngoscope,2001,111(2):356-358.
11. Leonardi A, Buonaccorsi S, Pellacchia V, et al . Maxillofacial prosthetic rehabilitation using extraoral implants[J].J Craniofac Surg,2008,19(2):398-405.
12. Wu YL, Schaaf NG. Comparison of weight reduction in different designs of solid and hollow obturator prostheses[J]. J Prosthet Dent, 1989,62(2):214-7.
13. Liu Yang,Hu Shuhai,Shi Xiaofang.Study of sound resonance for maxillary defect patients restored with high open hollow obturators.Journal of Dalian Medical University.1995;17(3):214-216.
14. Aramany MA,Drane JB.Effect of nasal extention sections on the voice quality of acquired cleft palate patients.J Prosthet Dent.1972,27(2):194-202.
15. Keyhani K,Scherer PW, Mozell MM.Numerical simulation of airflow in the human nasal cavity. J Biomech Eng.1995;117(4):429-41.

2. Objectives and content of the Research and the key scientific issues

Objectives

1) Compare the nasal respiratory function and the physiological data of the pulmonary ventilation volume of patients with maxilla defects so as to explain the reasons for clinical symptoms preliminarily.
2）Analysis on the structure change of the upper respiration tract before and after placing of maxillary prosthesis is performed by computational fluid dynamics in order to shed light on the pathophysiology mechanism of clinical respiratory symptom.
3）Measure the respiration parameters of the emulated upper respiration tract using the advanced flow field,pressure and acoustic testing methods,verify the reliability of numerical simulation of flow field and summerize the basic rule(law) of respirational flow.
4）Make an understanding of sound transmission caused by variation of airflow in the altered vocal resonance cavity through fluid and acoustic simulation in order to discover the factors which caused sound change and verify that sound is improved after maxillary rehabilitation.

Research contents

1) Pulmonary function, nasal sound reflection and nasal resistance of unilateral maxillary defect patients with/without obturator treatment should be tested to acquire nasal breathing changes and basic physiological data of postoperative patients, while providing appropriate physiological parameters for numerical simulation.
2) Analyze the CT data of patient before and after surgery to judge preliminarily the altered structure of upper respiration tract.Reconstruct and simulate numerically the upper respiration

tract before and after surgery for unilateral maxillary tumor patient.Analyze the aerodynamics parameters(velocity,pressure and airflow distribution trend) for patients after surgery.Design obturator and build the upper respiration tract model after rehabilitation according to the defected cavity of patients and analyze the aerodynamic law of airflow in the rehabilitated upper expiration tract and explain the pathophysiology mechanism of respirational related clinical symptoms.
3) Make the emulated model of the upper respiration tract using the rapid-prototyping technique and test aerodynamic physical parameters such as velocity ,pressure and so on .Analysis were done in an effort to get the law of velocity field distribution,pressure distribution and sound transmission law in the emulated model.Meanwhile,the results of the measurements can further verify the reliability of simulation of the airflow field.
4) Based on the coupling of the airflow field and acoustic field, analyze acoustic parameters in the resonance cavity and far field after surgery and rehabilitation respectively(sound pressure,frequency,acoustic pressure amplitude during each band,far field frequency amplitude variations,acoustic radiation etc),infer how the sound changes during its transmission in the resonance cavity.Analyze the trend of energy loss during the transmission of sound wave to supplement the results of airflow field and explore the reason why sound is changed .

3 Research programs to be taken and feasibility analysis.

Research procedure

1) nasal respiratory function and pulmonary function tests of maxillary defect patients before /after obturator treatment (15-20patients)

To evaluate the respiration function by choosing 15-20 patients with unilateal maxillary defect because of surgical resection. The geometric shape of the nasal cavity, cross-sectional area, volume and pressure breathing index were evaluated using acoustic rhinometry and rhinomanometry. A pulmonary function test system (CHESTAC-8800-D, Japanese CHEST Corporation, Japan) was used to measure SVC and FVC.The gold standard re-breathing method (helium dilution method) recommended by the American Thoracic Society was used in the FRC examination.
All patients were treated by a single maxillofacial prosthodontist to rehabilitate the oral defects with maxillary obturators. The measurements were performed after the initial insertion of the obturator after the unified standard evaluation was settled and after the obturator initially inserted. The evaluation standards were as follows: 1) the obturator was well adapted to the defect and 2) the retention was sufficient. The obturator had stable occlusion with the mandibular teeth. No obvious pain on mastication existed, and when the patient drank water while sitting upright, no fluid flowed from the patient’s nares. The following inclusion criteria were applied: no history of chronic diseases of the upper respiratory tract, no acute infections of the upper respiratory tract in the previous one month, and no partial medication history of the nasal cavity. Female patients who were pregnant or menstruating were excluded. Any patients with an incomplete nasal septum was also excluded through clinical examinations and imaging studies. all data were presented as the mean ± standard deviation (SD) to describe the data distribution. The one-sample

Kolmogorov-Smirnoov test was applied to the data distribution related to the prosthesis placement (before or after), and the normal distribution data were analyzed with a paired t-test, while the non-normal distribution data were analyzed with the Wilcoxon signed-rank test, and $P<0.05$ was considered to be statistically significant.

2) Three-dimensional reconstruction and gas flow numerical simulation of the respiratory tract of postoperative unilateral maxillary defect patients (3-5cases)

(1) establish the 3-D numerical model of the upper respiratory tract

①CT scanning

The patients were scanned with a spiral CT scanner (GE Light Speed 16, GE Medical Systems, USA), which extended from the supraorbital margin to the cricoid cartilage and had a slice thickness of 1.25 mm. The total slices were captured and stored as DICOM images on a CD-ROM.

②Establish numerical model

Then, the original 3-D model of the patient's head was reconstructed by stacking the DICOM images using Mimics software (Materialize NV, Leuven, Belgium). Numeric simulation was performed using CFD software (ANSYS12.0–CFX, ANSYS).Import the original 3-D Model into ANSYS ICEM CFD for trimming and mesh editing process,after that, establish tetrahedral volume mesh model; define the respiratory physiology entrances in accordance with respiratory Physiology law, then the three-dimensional numerical model of the upper respiratory tract will be built.

(2) numerical simulation of the 3-D model

①Boundary conditions and parameters are defined

The RNG-based κ-εturbulence model was used in the study. applying a standard atmospheric pressure at the anterior nostril .The boundary conditions were defined as follows: the pressure at nostrils was defined as atmospheric and airflow was defined at the level of larynx. Apply the CFD methods for simulating and unsteady calculating of the entire respiratory cycle.

②numerical simulation

Following results should be aquired:(1) the whole airflow trends of upper respiratory tract in patients; (2)different cross-sectional area, volume; velocity, pressure distribution characteristics at different anatomical levels of the ill side and the healthy side; (3) gas flow proportional characteristicsof the respiratory tract at different anatomical levels; (4) eddy kinetic effects; (5)comparative analysis on the respiratory breathing pattern airway resistance and anatomical structural changes of the patients before/after surgery.

3) Numeric smulation of upper expiration tract after CAD rehabilitation.(3-5cases)

(1)design the prosthesis

Reconstruct the upper respiration model of patient with unilateral maxillary tumor based on the CT images and design hollow obturator with reference to the defected cavity、the nasal structure morphology and airflow characteristics .

(2) Simulate numerically the upper expiration tract after rehabilitation.

Build the rehabilitated upper expiration tract 3-D model, draw the body grids and define the inlet and outlet,the numerically simulation of the upper expiration tract after rehabilitation was done.

Compare the respiration mode,resistance of respiration tract,volumn of flow,vorticities, structure morphology,airflow in the defected cavity in order to understand the effect of prosthesis on respiration.

4) Simulate the respiration of the upper respiration tract in vitro ,verify the reliability of flow field simulation.

(1) Make the emulated model of the upper respiration tract (1 case)

Make resin respirational model of the healthy people using SLA method.

(2)control the respiration process

Simulate the respiration process through the synthetic jet method, and control the influx of respiration by adjusting the vibrating frequencies of synthetic jet.

(3) Assessment of airflow field 、pressure and acoustic parameters

Assess the respiration airflow field of emulated model for maxillary defected patient in vitro by PIV.Test the pressure of respiration tract using high frequency dynamic pressure sensor,and assess the acoustic characteristics of far field using the acoustic microphone.

5) Sound field simulation for unilateral maxillary defected patient after surgery and rehabilitation(3-5cases)

According to the clinical speech test research and related literature, vowel /i:/ is most likely to be influenced by the structure change of acoustic tract.We asked the patient to pronounce /i:/ during the spiral CT scanning procedure.Reconstruct the 3D acoustic model in the Mimics software based on the CT data. Draw grids and define the inlet and outlet of the acoustic tract model and import it into acoustic analyzing software.Select several characteristic frequencies and analyze the sound pressure ,frequency band and sound radiation of the outer far field so as to explore the reason why sound is changed after surgery.
