# Supplementary material for: The Influence of Obturators on the Respiration of Patients with Maxillary Defects: A Clinical Study
Source: PLoS One. 2015 May 26;10(5):e0127597. doi: 10.1371/journal.pone.0127597 (PMC4444186; doi:10.1371/journal.pone.0127597)
Supplement: S2 Protocol — (PDF) [file pone.0127597.s002.pdf]

申请代码	
受理部门	
收件日期	
受理编号	

检查保护

国家自然科学基金

申 请 书

（２０１１ 版）

您现在不能检查保护文档或打印文档，请根据以下三个步骤操作：

1)如果您是 Word2000,word XP, word 2003 或以上版本用户，请把 Word 宏的安全性设为:"中"

方法: Word 菜单->工具->宏->安全性->安全级,设置为"中"

(如果您是 Word97 用户，继续执行以下步骤)

(如果您是 Office2007 用户，点击 word 左上角"安全警告"处"选项"中的"启用此内容")

2)关闭本文档，重新打开本文档

3)点击"启用宏"按钮，即可开始填写本文档或打印了

申报日期： 2011年3月1日

国家自然科学基金委员会

基本信息

申请人信息							
姓名	焦婷	性别	女	出生年月	1972 年 4 月	民族	汉族
学位	博士	职称	副教授	每年工作时间（月）	5		
电话	021-23271699-5693	电子邮箱	jiao_ting@hotmail.com				
传真		国别或地区	中国				
个人通讯地址	上海市制造局路 639 号						
工作单位	上海交通大学 /医学院附属第九人民医院						
主要研究领域	口腔修复学；口腔颌面赝复学						

依托单位信息			
名称	上海交通大学		
联系人	张艳	电子邮箱	jhk-jyb@sjtu.edu.cn
电话	021-34206809-182	网站地址	www.sjtu.edu.cn

合作研究单位信息
单位名称
[在此录入修改]
[在此录入修改]

项目基本信息			
项目名称	基于数值模拟的上颌骨缺损患者呼吸与发音变化机制研究		
资助类别	青年科学基金项目	亚类说明	
附注说明			
申请代码	H1408:牙缺损、缺失及牙颌畸形的修复与矫治		
基地类别			
研究期限	2012 年 1 月 — 2014 年 12 月	研究属性	应用基础研究

摘要

（限 400 字）：上颌骨缺损造成上呼吸道解剖结构改变，引起患者呼吸不适与发音不清等临床症状，严重影响患者生活质量。阻塞器赝复体能有效地改善这些临床症状，但有些患者的改善效果仍然不够理想。目前国内外对上颌骨缺损后呼吸和发音变化机制的研究较少，使临床医师不能正确地判断修复效果、有效地改进赝复体制作。我们前期的研究证实了数值模拟技术能定量地分析上呼吸道的气流变化规律，为颌面赝复研究提供了新思路。本课题从患者上呼吸道通气功能变化的临床测试出发，将流体力学数值模拟与先进的实验流场测试相结合，研究上颌骨缺损及修复后患者的上呼吸道气体流动特点；通过流场和声场的耦合，探索修复前后发声共鸣腔结构改变对声传播的影响，研究呼吸道流场与声场的能量变化的关系，从而基本阐明上颌骨缺损患者呼吸与发音的变化机制，完善颌面赝复学的基础理论，为指导临床医生合理设计赝复体，指导患者术后功能康复提供理论依据。

关键词（用分号分开，最多 5 个）：上颌骨缺损；数值模拟；呼吸；发音；流场与声场

项目组主要参与者（注：项目组主要参与者不包括项目申请人）

编号	姓　名	出生年月	性别	职 称	学 位	单位名称	电话	电子邮箱	项目分工	每年工作时间（月）
1	吴亚东	1980-5-21	男	讲师	博士	上海交通大学	021-34206320	yadongwu@sjtu.edu.cn	流声场分析指导	4
2	熊耀阳	1977-9-14	女	主治医师	博士	上海交通大学	021-23271699	yaoyangx@hotmail.com	赝复体与声学图像处理	4
3	陈丽萍	1961-7-30	女	教授	学士	上海交通大学	021-23271699	chenliping118@citiz.net	临床呼吸测试	4
4	陈晓波	1979-9-15	男	博士后	博士	上海交通大学	021-34206771	xiaoboc@sjtu.edu.cn	体外实验流场测量分析	4
5	钱玉梅	1985-12-10	女	医师	硕士	上海交通大学	021-23271699	graceyumei@163.com	三维建模与流场分析	6
6	董晛	1986-3-24	女	硕士生	学士	上海交通大学	021-23271699-5693	124791570@qq.com	实验流场中的呼吸模拟	8
7	盖德倩	1984-10-7	女	硕士生	学士	上海交通大学	021-23271699	deqiangai330@hotmail.com	三维建模与声学分析	8
8	王昊	1986-7-3	男	硕士生	学士	上海交通大学	021-34205635	wanghao257@sjtu.edu.cn	流场与声场计算模拟	8
9	[在此录入修改]									

总人数	高级	中级	初级	博士后	博士生	硕士生
9	2	2	1	1		3

说明：　高级、中级、初级、博士后、博士生、硕士生人员数由申请人负责填报（含申请人），总人数由各分项自动加和产生。

经费申请表

（金额单位：万元）

科目	申请经费	备注（计算依据与说明）
**一. 研究经费**	21.0000	
1. 科研业务费	12.0000	
（1）测试/计算/分析费	8.0000	患者鼻声、鼻阻力及肺通气功能的临床测试；三维重建、流场与声场模拟、实验模拟等
（2）能源/动力费	0.5000	水电气、交通、通讯费等
（3）会议费/差旅费	2.0000	2012 年中华口腔学会会议、2013 年全国生物力学会议
（4）出版物/文献/信息传播费	1.5000	购买参考书籍、订阅杂志；文章查新、检索；版面费等
（5）其他		
2. 实验材料费	7.0000	
（1）原材料/试剂/药品购置费	5.0000	赝复体及仿真模型制作的相关材料；仿真模型内流体系统液体及控制相关的物理电学原件等
（2）其他	2.0000	CT 扫描；临床测试仪器中耗材的补充，氦气等；先进流场、声场测试仪器的的校准与维护
3. 仪器设备费	2.0000	
（1）购置	2.0000	气体流场和声场数据处理设备
（2）试制		
4. 实验室改装费		
5. 协作费		
**二. 国际合作与交流费**	2.8000	
1. 项目组成员出国合作交流	2.8000	ISMR 会议、ICP 等会议
2. 境外专家来华合作交流		
**三. 劳务费**	2.8000	直接参加项目研究的研究生的劳务费用
**四. 管理费**	1.4000	项目组织实施管理
**合　　计**	28.0000	

与本项目相关的其他经费来源	国家其他计划资助经费	0.0000
	其他经费资助（含部门匹配）	0.0000
	**其他经费来源合计**	0.0000

申请者在撰写报告正文时，请遵照以下要求：

1、 请先选定"项目基本信息"中的"资助类别"，再填写报告正文；

2、 在撰写过程中，不得删除系统已生成的撰写提纲（如误删可点击“查看报告正文撰写提纲”按钮，通过"复制/粘贴"恢复）；

3、 请将每部分内容填写在提纲下留出的空白区域处；

4、 本要求将作为申请书正文撰写是否规范的评判依据，请遵照要求填写。

查看报告正文撰写提纲

报告正文

青年科学基金项目申请书撰写提纲

（一）立项依据与研究内容（4000-8000 字）：

1. **项目的立项依据**（研究意义、国内外研究现状及发展动态分析，需结合科学研究发展趋势来论述科学意义；或结合国民经济和社会发展中迫切需要解决的关键科技问题来论述其应用前景。附主要参考文献目录）

上颌骨缺损是口腔颌面部缺损修复中最常见、发生率最高的缺损，约占颌面部缺损患者的 58.8%[1]。上颌骨缺损造成患者严重的牙缺损与颅颌面畸形，包括：鼻黏膜缺损、口鼻腔相通、软硬腭功能破坏、发音闭合环与共鸣腔破坏等，并在临床上出现鼻分泌物堆积、缺损侧呼吸不适以及发音不清等功能障碍，对患者的心理和生活质量造成很大的危害。阻塞器赝复体是修复上颌骨缺损最简便有效的治疗方法[2-5]，能基本恢复患者的呼吸和发音等生理功能[4]。但是，有些患者修复后仍然有呼吸不畅、耳鸣和发音不够清晰等症状。为什么患者术后会出现一系列相同的临床症状？为什么赝复治疗后不是所有的患者都会出现上述不适？

目前国内外对上颌骨缺损后呼吸和发音变化机制的研究较少，现有的颌面赝复理论不能对这些临床症状和问题做出明确的解释。因此，明确上颌骨缺损及赝复治疗后患者的上呼吸道呼吸功能变化和发音变化规律，有利于指导颌面赝复医师对修复效果做出正确的判断、有效地改进赝复体的制作，有利于医师与患者进行良好的交流，使患者了解疾病对机体造成的影响和修复的效果，从而积极地配合康复治疗，同时为提高颌面赝复临床治疗水平提供理论依据。

由于呼吸道的复杂结构，真实的反映其生理变化的定性与定量研究具有一定难度。近年来，呼吸系统数值模拟成为研究热点[6]，主要集中于正常、异常鼻腔疾病或阻塞性睡眠呼吸暂停低通气综合症（OSAHS）等病理性上呼吸道疾病。Lindemann[7-11]等通过对单侧根治性鼻窦手术和阻塞性呼吸暂停低通气综合症(OSA)鼻腔气流进行数值模拟分析，认为上呼吸道的 CFD 数值模拟模型有助于研究疾病的发病机理和预测效果。在国内，课题组前期的研究[12]及孙秀珍[13-14]、郭宇峰[15]等对鼻腔或上呼吸道的流体力学模拟进行相关研究，证实重建鼻腔与上呼吸道结构和数值模拟方法可行。但是这些研究结果多限于得到正常或异常鼻腔、上呼吸道疾病的气体流动趋势，而对于上颌骨缺损及赝复修复后整个上呼吸道（包括双侧鼻腔、咽、喉）的呼吸模式变化及基础生理数据，未见文献报道。课题组前期对 2-3 例患者的鼻声反射、鼻阻力的初步测试，发现其相关指标发生了变化，而戴用阻塞器后指标又有所回升。同时在对单侧上颌骨缺失患者的呼吸道三维建模及流体力学的预初研究，发现缺损侧鼻腔和健侧鼻腔气流模式显著不同。课题组认为应该对这类患者术后及赝复后整个上呼吸道解剖结构、呼吸气流变化以及发声变化的基本规律进行研究，从而揭示术后与赝复后出现共同临床症状的病生机制。

人的语言是一个结合了上消化道和呼吸道的偕同功能。人类的发音几乎都是用呼出的气流来形成的，发声生理器官包括三个部分：（1）肺和相关肌群等活动提供发声所必要的能量，（2）构成发声的动力器官包括声带振动构成声源与喉发出较弱的单调的基音，（3）经过口腔、鼻腔及咽腔共同组成的共鸣腔形成空气的振动，选择性地过滤和抑制某些频率，才能形成清晰悦耳的声音[16-17]。上颌骨切除的患者，构音结构（嘴唇、舌头、面颊、上腭）中的上腭受到影响（切除破坏），更重要的是其共鸣腔的结构发生明显改变。患者由于软、硬腭缺损，造成上下共鸣腔相通，造成口腔与鼻腔的压力改变，口腔和鼻腔内发出的声音比例即气流通过的比例有所改变，从而影响语音的质量。通常，这种发音的变化表现为鼻腔共鸣太多、鼻腔共鸣太少、或两者混合存在。课题组通过多年来临床经验总结，认为阻塞器赝复体封闭了口鼻相通的硬腭，并在一定程度上恢复了软腭封闭功能，从而改变了共鸣腔的形态和压力，使声音传导过程发生了改变。

目前对于颌骨缺损患者声学的研究主要是采用计算机语音分析系统分析语音清晰度及共振频率。课题组在过去的研究中[4]发现阻塞器对患者元音发音的改善作用在一个月时趋于稳定，尤其对/a/、/i/元音的舌、腭定位起到重要作用。Arigbede 等[18-19]也发现依次戴用阻塞器可较好的改善患者的语音清晰度。但上述研究是对临床现象的客观评

价，而对于气流在术后改变的共鸣腔内如何传导，气流的变化造成声音传播时发生了哪些改变，阻塞器修复后气流通过的比例如何变化以恢复患者的语音，整个过程中声波能量是否有消耗，对患者的发音改变有否作用，除了课题组提出的舌、腭定位作用以外，未见相应报导。

课题组通过对上颌骨切除患者上呼吸道气体流场的预初计算，发现上颌骨切除患者的气体流场类似于空腔流动效应[20-21]。由此，课题组提出假设：这类患者的发音障碍不是缺少气流，而是由于能量的无效利用浪费了气流，气流在共鸣腔内声压幅值和声音频率可能发生了变化。阻塞器赝复体是通过改变共鸣腔结构形态，减少空腔效应，部分恢复气流在声道内传导的声压和频率，从而起到恢复语言清晰度的作用，这一假想需要进一步声场的数值模拟得以论证。

本项目在过去对颌面缺损研究的基础上[22-25]，从患者上呼吸道通气功能变化的临床测试出发，首次建立单侧上颌骨缺损以及阻塞器修复后上呼吸道三维数字化模型，进行上呼吸道流场数值模拟，定量分析气体流场参数（速度、压力和气流分布趋势等）的变化，阐明这类患者由于上呼吸道解剖结构改变所引起的气流变化规律；应用快速成型技术制作上呼吸道气流仿真模型，并通过先进的流场测试技术、动态压力测试技术以及声学测试技术相结合，对上颌骨缺损患者的呼吸流场进行实验测量分析，总结呼吸流动的基本规律，并进一步验证流体力学流场模拟的可靠性；通过呼吸气流的流场和声场的耦合分析研究，运用声场数值模拟，定量分析术后及修复后患者共鸣腔内部的参数（声压、频率及各个频段上的声压幅值）及远场效应变化（远场声辐射情况及频率变化），揭示气流变化引起声波能量变化的情况，进一步阐述上颌骨缺损后发音变化的原因；为基本阐明上颌骨缺损患者呼吸与发音变化规律，完善颌面赝复学理论打下基础。

主要参考文献

1. 赵铱民.颌面赝复学[M].西安:世界图书出版公司,2004:1-5.
2. Ortegon SM, Martin JW, Lewin JS.A hollow delayed surgical obturator for a bilateral subtotal maxillectomy patient: a clinical report [J]. J Prosthet Dent, 2008,99(1):14-18.
3. Barnouti L. Caminer D.Maxillary tumours and bilateral reconstruction of the maxilla [J].ANZ J Surg, 2006,76(4):267-269.
4. 邢国芳,焦婷,孙健,蒋永林.单侧上颌骨切除术后赝复体修复患者的语音评价[J].上海口腔医学,2005,14(4):352-354.

5. Itsuki Murase.In-vivo modal analysis of maxillary dentition in a maxillectomy patient wearing buccal flange obturator prostheses with different bulb height designs[J]. Nihon Hotetsu Shika Gakkai Zasshi, 2008, 52(2):150-159.
6. Keck T, Lindemann J. Simulation and air-conditioning in the nose. Laryngorhinootologie. 2010 May;89 Suppl 1:S1-14.
7. Lindemann J,Brambs HJ,Keck T, Wiesmiller KM, Rettinger G. Numerical simulation of intranasal airflow after radical sinus surgery [J]. Am J Otolaryngol，2005,26(3):175-180.
8. Mylavarapu G, Murugappan S, Mihaescu M, Kalra M, Khosla S, Gutmark E. Validation of computational fluid dynamics methodology used for human upper airway flow simulations[J]. J Biomech, 2009, 42(10):1553-1559.
9. Kastl KG,Rettinger G,Keck T.The impact of nasal surgery on air-conditioning of the nasal airways. Rhinology[J], 2009,47(3):237-241.
10. Sung SJ, Jeong SJ, Yu YS, Hwang CJ, Pae EK. Customized three-dimensional computational fluid dynamics simulation of the upper airway of obstructive sleep apnea [J]. Angle Orthod,2006,76(5):791-799.
11. Jeong SJ, Kim WS, Sung SJ. Numerical investigation on the flow characteristics and aerodynamic force of the upper airway of patient with obstructive sleep apnea using computational fluid dynamics [J].Med Eng Phys,2007,29(6):637-651.
12. 钱玉梅，陈丽萍，吴亚东，焦婷. 人体上呼吸道三维数值模型的建立与气体流场数值模拟分析[J].上海口腔医学,2010,19(2):164-168.
13. 王吉喆,张军,孙秀珍,刘迎曦. 鼻腔流场数值模拟与鼻声反射相关性研究[J]. 医学与哲学(临床决策论坛版),2007,28(5):52-54.
14. 于申, 刘迎曦, 孙秀珍, 苏英锋. 鼻腔气道结构对鼻腔加温加湿功能影响的数值模拟[J]. 医用生物力学,2010,(06):444-448.
15. 郭宇峰,张宇宁,刘树红, 卢晓峰, 朱敏, 陈学明, 陈广.鼻腔计算机流体力学模拟及与鼻声反射和鼻阻力计相关研究[J]. 上海交通大学学报(医学版),2009,29:845-849.
16. 姜泗长,顾瑞主编.言语语音疾病学[M].北京:科学出版社,2005.p19-32
17. 杨式麟.嗓音医学基础与临床[M].沈阳:辽宁科学技术出版社,2001.p88-121
18. Arigbede AO, Dosumu OO, Shaba OP, Esan TA. Evaluation of speech in patients with partial surgically acquired defects pre and post prosthetic obturation[J]. J Contemp Dent

Pract,2006,7(1):89-96.
19. Turkaslan S, Baykul T, Aydin MA, Ozarslan MM. Articulation performance of patients wearing obturators with different buccal extension designs[J]. Eur J Dent, 2009, 3(3):185-190
20. 马明生,张培红,邓有奇,吴晓军. 超声速空腔流动数值模拟研究[J].空气动力学学报,2008,26(3):388-392.
21. 李晓东,刘靖东,高军辉. 空腔流激振荡发声的数值模拟研究[J].力学学报,2006,38(5):559-606.
22. Jiao T, Zhang F, Huang X, Wang C. Design and Fabrication of auricular prostheses by CAD/CAM System[J]. Int J Prosthodont,2004,17(4):460-463.
23. Sun J, Jiao T, Tie Y, Wang DM. Three-dimensional finite element analysis of the application of attachment for obturator framework in unilateral maxillary defect[J]. J Oral Rehabil,2008,35(9):695-699.
24. 洪凌斐,孙健,焦婷,张保卫, 铁瑛, 王冬梅.单侧上颌骨缺损修复三维有限元模型的建立及力学分析[J].上海口腔医学,2006,15(4):403-406.
25. 焦婷, 孙健,洪凌斐,张富强, 铁瑛, 王冬梅.附着体应用于单侧上颌骨缺损修复的三维有限元分析[J]. 上海口腔医学, 2006,15(5): 370-374.

2. 项目的研究内容、研究目标，以及拟解决的关键科学问题。（此部分为重点阐述内容）

研究目标

1) 比较上颌骨缺损患者鼻腔呼吸功能与肺通气量生理数据的变化，初步解释临床症状产生的原因。
2) 流体力学分析上颌骨缺损赝复修复前后上呼吸道结构变化对呼吸功能的影响，阐明患者出现鼻腔及呼吸等临床症状产生的病生机制。
3) 应用先进的流场、压力以及声学测试手段对上呼吸道仿真模型体外模拟上呼吸道的呼吸情况进行测量，总结呼吸流动的基本规律，并验证流场数值模拟的可靠性。
4) 通过流场和声学模拟，了解在结构改变的发声共鸣腔中，气流变化所引起的声

音传播的变化，探索患者发音变化的原因以及赝复治疗对发音改善的作用。

研究内容

1) 测试单侧上颌骨缺损及赝复体修复后患者的肺通气功能、鼻声反射和鼻阻力，获取患者术后呼吸与鼻腔变化的基本生理数据与变化情况，同时为数值模拟提供相应的生理参数。

2) 分析患者手术前后CT数据，初步判断上呼吸道结构的变化情况；分别对单侧上颌骨肿瘤患者手术前后的上呼吸道进行三维重建和气体流场数值模拟，分析患者术后上呼吸道的气流动力学变化规律（速度、压力和气流分布趋势等）；根据患者缺损腔结构特征，设计阻塞器，建立赝复修复后的上呼吸道三维模型，分析赝复体修复后上呼吸道的气流动力学变化规律，阐明患者出现鼻腔及呼吸等临床症状病生机制。

3) 应用快速成型技术制作上呼吸道气流仿真模型，使用先进的流场测试设备，如时间解析粒子图像速度仪（Time Resolved Particle Image Velocimetry）对体外模拟上颌骨缺损患者的呼吸流场进行精细化测量，使用高频响动态压力传感器测量呼吸道的压力变化，使用声学麦克风测量远场的声音特征，从而获得仿真模型的速度场分布、压力分布以及声音传播规律。同时，测量的结果可以进一步验证流体力学气场模拟的可靠性。

4) 基于气体流场和声场的耦合，分析上颌骨缺损与阻塞器修复后，共鸣腔内部及远场声学参数的变化（声压、频率、各频段声压幅值的变化、远场频域变化、声辐射等），推断共鸣腔中声传播变化模式。分析在声波传播过程中，由于气流变化造成的能量损耗趋势，对流场的结果进行补充说明，探索患者发音变化的原因。

拟解决的关键问题

1）复杂腔体三维重建与流场数值模拟生理边界条件的定义。本项目组研究人员经过专门的医学图像成像和处理软件的培训，掌握了复杂腔体的三维建模。通过大量国内外文献阅读和反复验证，已基本确定这类复杂腔体患者中气体流场数值模拟边界条件的定义。通过前期正常人体上呼吸道数值模拟计算，验证了生理边界条件定义的可靠性。

2）上呼吸道仿真模型的制作与体外上呼吸道流场模拟。课题组曾多次完成复杂曲面的快速成型，对可用于流体模拟的仿真模型制作已找到合适的材料和方法。通过前期的文献检索，已找到方法进行呼吸模拟控制，通过先进的测试手段测量人体呼吸的流动情况。

3）复杂空腔的流场与声场的耦合分析。通过分析复杂腔体的气体流场预初数据，课题组成员发现这类患者上呼吸道的气体流动类似于空腔流动效应，为这类复杂空腔流动提供理论上的可行性。

3. 拟采取的研究方案及可行性分析。（包括有关方法、技术路线、实验手段、关键技术等说明）

研究方案

1) 上颌骨缺损患者赝复体修复前后鼻腔呼吸功能与肺通气功能测试（15-20名）

运用鼻阻力计与声反射仪测定鼻腔几何形态、横截面积、容积与压力等呼吸指标，并与数值模拟结果比较，验证气体流场数值模拟结果的可靠性。（具体实验方法参照《鼻科学》第二版，上海科学技术出版社）

运用肺功能检测仪测定量肺的通气功能：肺活量(SVC)、用力肺活量(FVC)，以及采用公认的“金标准”重复呼吸法（氦气跟踪法或称平衡法）精确测量功能残气量（FRC）。以期为流体力学分析的数值结果提供生理学依据。（具体实验方法参照《临床肺功能》，人民卫生出版社）

2) 单侧上颌骨肿瘤患者术后上呼吸道三维重建与气体流场数值模拟（3-5名）

（1）建立单侧上颌骨缺失的上呼吸道三维数值模型

①CT扫描

采用GE Light speed 16螺旋CT（GE medical system，USA），扫描时患者取仰卧位，扫描范围自眶上缘开始到颈根处为止，采取轴位扫描，层厚 1.25mm，扫描的二维断层

图像以 DICOM 存储格式输出保存。

②数值模型的建立

Mimics 软件读取患者的 CT 扫描图像（包含冠状位、横断面、矢状位不同层面图像），经图像分割处理快速生成单侧上颌骨缺失的上呼吸道三维表面模型；模型导入 ANSYS ICEM CFD 进行模型的修整和体网格划分编辑处理的操作，建立四面体体网格模型；按照生理呼吸规律定义上呼吸道出入口，建立单侧上颌骨缺失的上呼吸道三维数值模型。

（2）单侧上颌骨缺失的上呼吸道三维数值模拟

①边界条件与参数定义

采用 RNG κ-ε 两方程湍流模型。前鼻孔施加一个标准大气压,喉腔下缘定义为气流量边界条件，四周壁定义为动态壁面边界条件。应用 CFD 方法对患者的上呼吸道整个呼吸周期进行非定常数值模拟计算。

②数值模拟

获取患者上呼吸道整体气流流动趋势；健侧与缺损侧不同解剖层面的横截面积、体积；速度、压力分布特征；呼吸道不同解剖层面气流量比例规律；涡动力学效应；对比分析上颌骨肿瘤患者术前和术后上呼吸道呼吸模式、呼吸道阻力与解剖结构变化特点等。

3) 单侧上颌骨肿瘤患者赝复修复后上呼吸道三维重建与气体流场数值模拟（3-5 名）

（1）赝复体设计

根据单侧上颌骨肿瘤患者术后 CT 图像，参照三维重建后的缺损腔、鼻腔结构形态特征，结合上呼吸道气流流动特点，设计中空阻塞器。

（2）赝复修复后的上呼吸道数值模型建立

依据图像分割处理功能，阻塞器分隔口鼻腔穿通，建立赝复修复缺损后的上呼吸道三维表面模型，经体网格划分和定义出入口，完成赝复修复后的上呼吸道三维数值模型。

（3）数值模拟

分析单侧上颌骨缺损患者赝复修复后与修复前相比，呼吸模式、呼吸道阻力、流量、涡量、结构形态、缺损侧气流流动改变情况等；了解赝复体在患者呼吸中的作用。

4) 体外模拟上呼吸道的呼吸，验证流场计算的可靠性

（1）上呼吸道仿真模型的制作（1 例）

在图像处理软件中，通过图像反求，将步骤 2 形成的上颌骨缺损模型反求生成 1:1 仿真的上呼吸道实体模型，应用立体印刷术（SLA 法）制作相应的透明树脂模型。

（2）呼吸过程控制

采用合成射流的方法模拟呼吸过程，通过调节合成射流的振动频率控制呼吸气流的流量。

（3）流场、压力以及声学参数测量

使用跟随性好的示踪粒子，采用时间解析粒子图像速度仪（Time Resolved Particle Image Velocimetry）对体外模拟上颌骨缺损患者的呼吸流场进行精细化测量，使用高频响动态压力传感器测量呼吸道的压力变化，使用声学麦克风测量远场的声学特征。

5) 单侧上颌骨缺损术后与赝复后的声场模拟（3-5 名）

(1) 模型导入

将步骤 2,3 中所建的缺损与赝复的三维模型导入声学模拟软件，确定软硬质结合的边界条件。将流体计算的结果导入声学模拟软件，准备流场与声场的耦合。

(2)声源选择

根据课题组过去临床语音测试研究与相关文献[4,16]，元音/a/、/i/；鼻腔共鸣音/m/、/n/、/ng/；齿擦音/s/；塞音/p/、/t/对患者发音影响较大。通过正常人的测试，获得这些声源所在的频率范围。

(3)上呼吸道共鸣腔内部的声学参数分析

将模型导入声学模拟软件，选用不同的声源频率，计算上述两模型的呼吸道共鸣腔内部的声压、频率以及各个频段上的声压幅值变化，判定声压脉动最强的频段；模拟在不同频段声源作用下，呼吸道外部远场的声压、频域变化及声辐射情况，以测定不同元音和辅音的频率在远场效果。分析在流场中涡量动力学效应与声场中声能传导方向、消减变化对声音传导的影响。

技术路线

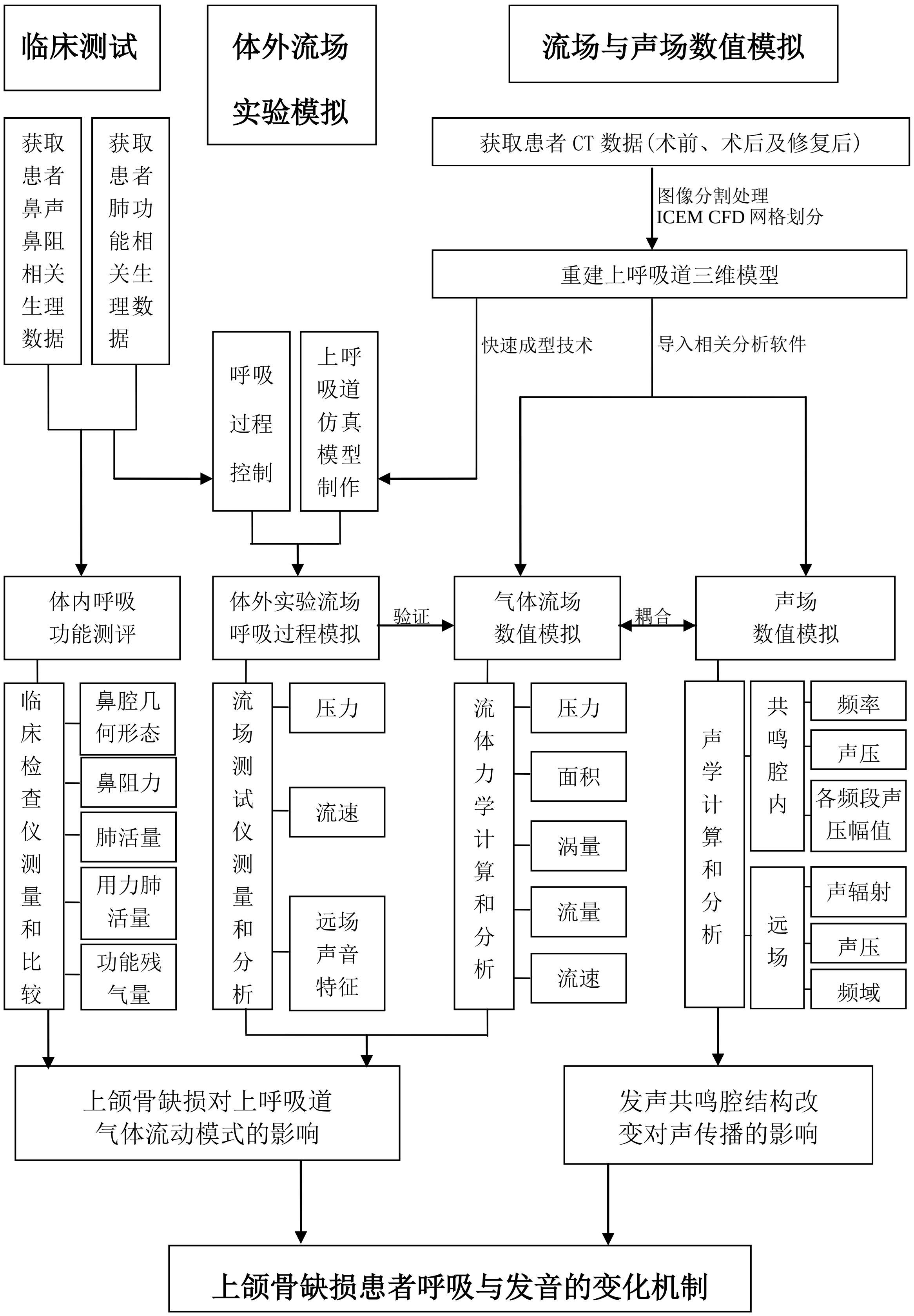
临床测试
体外流场实验模拟
流场与声场数值模拟
获取患者鼻声鼻阻相关生理数据
获取患者肺功能相关生理数据
获取患者 CT 数据(术前、术后及修复后)
图像分割处理
ICEM CFD 网格划分
重建上呼吸道三维模型
快速成型技术
导入相关分析软件
呼吸过程控制
上呼吸道仿真模型制作
体内呼吸功能测评
体外实验流场呼吸过程模拟
验证
气体流场数值模拟
耦合
声场数值模拟
临床检查仪测量和比较
鼻腔几何形态
鼻阻力
肺活量
用力肺活量
功能残气量
流场测试仪测量和分析
压力
流速
远场声音特征
流体力学计算和分析
压力
面积
涡量
流量
流速
声学计算和分析
共鸣腔内
频率
声压
各频段声压幅值
远场
声辐射
声压
频域
上颌骨缺损对上呼吸道气体流动模式的影响
发声共鸣腔结构改变对声传播的影响
上颌骨缺损患者呼吸与发音的变化机制

可行性分析

1) 实验设计合理。上颌骨缺损后上呼吸道结构改变而引起功能改变并出现相关的临床症状和体征。课题从临床测试出发，以气体流场数值模拟为依据，结合上呼吸道仿真流体模型的实验流场测试，获得上呼吸道气流动力学变化特征，然后在此基础上通过气场与声场的耦合，进入声场研究声音传导的声幅频率和频域的变化，可以较全面的反映上颌骨缺损患者修复前后的呼吸与发音变化规律，最终揭示患者出现相同临床症状以及临床治疗改善症状的机理。
2) 研究方案的可行性。已有的文献及课题预初实验都证实了计算流体力学技术数值模拟鼻腔或上呼吸道的可行性；而工程学在航空航天及船舶领域声场空腔流动效应的理论研究与技术方法可以借鉴到医学复杂腔体声场传导的理论研究中；结合了先进的流场测试技术和声场测试技术，能较真实的模拟上呼吸道复杂气体流动和声学特性的变化。研究方案层层推进，使最终阐明上颌骨缺损患者的呼吸和发音变化机制得到可能。
3) 研究对象充足。上海第九人民医院每年上颌骨切除患者超过 100 例，患者在术前、术后 3 个月及 1 年都需要做 CT 或磁共振检查，因此可以获得足够的 CT 图像数据。
4) 计算软件与硬件设施：第九人民医院拥有先进的鼻声反射仪、鼻阻力计、肺功能测量仪、16 排螺旋 CT 与 MRI，以及图像处理软件 MIMICS，Geomagic。上海交大航空航天学院已拥有流体力学计算相关软件，最新购置了声学软件 ACTRAN, 以及时间解析粒子图像速度仪、高频响动态压力传感器、声学麦克风等先进的硬件设施，同时交大机械学院有先进的快速成型设备，保证实验用仿真模型的制作，可以保证整个实验的顺利计算与运行。
5) 课题组团队结构合理，涵盖了本课题所需的各学科的人才。在过去的研究中课题组成员互相合作，建立了良好的团队合作。

4. 本项目的特色与创新之处。

1) 首次关注到上颌骨缺损与赝复修复后患者的上呼吸道通气功能的变化并获得相关的数据。
2) 首次将流体力学数值模拟与先进的实验流场测试相结合，应用于上颌骨缺损及

修复后患者的上呼吸道气体流动结构的研究中，阐述患者赝复修复前后呼吸变化规律。

3) 首次通过流场和声场的耦合，寻找单侧上颌骨肿瘤患者修复前后发声共鸣腔结构与声传导变化规律，将流场中涡的形成与声场中能量的变化相结合，对上颌骨缺损患者赝复修复前后的呼吸与发音变化进行探讨。

5. 年度研究计划及预期研究结果。（包括拟组织的重要学术交流活动、国际合作与交流计划等）

年度研究计划

2012.1-2012.12

制定研究对象入选标准，依照标准选择上颌骨缺损患者作为研究对象，赝复体修复治疗，测量研究对象修复前后鼻腔几何形态及鼻阻力的生理数据，比较修复前后鼻呼吸功能的改变；测试研究对象修复前后的肺通气功能，为后续研究提供生理数据值。

基于手术前后 CT 图像数据，重建上颌骨缺损患者上呼吸道三维模型，并进行气体流场数值模拟分析。（参加 ISMR 会议）

2013.1-2013.12

应用快速成型技术制作比例 1：1 的上呼吸道仿真模型；呼吸过程的模拟和相关参数的控制；使用时间解析粒子图像速度仪（Time Resolved Particle Image Velocimetry）对体外模拟上颌骨缺损患者的呼吸流场进行精细化测量；使用高频响动态压力传感器测量呼吸道的压力变化；使用声学麦克风测量远场的声音特征。(参加 ICP 会议)

2014.1-2014.12

数值模型导入相关软件，选择合适声源音频；耦合气体流场和声场，数字模拟上颌骨缺损与阻塞器修复后，共鸣腔内部及远场声学参数的变化，分析共鸣腔中声传播变化模式与能量变化；论文撰写，结题鉴定。(参加 IADR 会议)

预期研究结果

1) 得到上颌骨缺损患者鼻腔几何形态、鼻阻力与肺通气功能的生理数据在修复前后的变化规律。

2) 基于流场模拟与声学模拟，解释上颌骨缺损造成的患者呼吸、发音改变的机制，以及赝复体改善患者呼吸、发音功能的机理。

3) 发表论文 4-6 篇，SCI 论文 1-2 篇。参加国内、国际会议 2-3 次。

4) 培养研究生 2-3 名。

（二）研究基础与工作条件

1、工作基础（与本项目相关的研究工作积累和已取得的研究工作成绩）

1. 上海交通大学医学院附属第九人民医院口腔修复科是上海市重点学科,在口腔颌面赝复治疗和康复方面处于全国领先地位,能为本项目提供足够的临床病例。现已筛选一定数量的上颌骨肿瘤患者 CT 图像数据。医院的呼吸科拥有经验丰富的临床医师以及先进的肺功能测试仪器，可以完成呼吸功能的测试与理论指导。

2. 项目负责人在美国加州大学洛杉矶分校专业系统学习了颌面赝复学的临床和理论知识，对上颌骨肿瘤的序列治疗，上颌骨肿瘤术后的口腔保健与颌面赝复领域积累了丰富的经验。课题组过去赝复领域的上颌骨缺损研究涵盖了生物力学、咀嚼语音功能、CT 和 MRI 三维重建在赝复治疗和康复中的应用，并多次在国内外杂志、会议上发表 SCI 论文。

3. 课题负责人与组员在三维重建与有限元力学分析、快速成型制作领域已经积累了近 10 年的经验，相关的三维重建和快速成型的文章发表在国际口腔修复杂志（Int J Pros 2004），三维有限元文章发表在口腔修复学杂志(J Oral Rehabil 2008)，上呼吸道流体力学分析文章发表在上海口腔医学杂志（2010），前期的上颌骨预初结果被 9th ISMR(国际颌面赝复学会议)收录并做大会英语发言（见附件 1），获得 6th IADR 中国分会杰出青年学者奖（见附件 2）。

4. 项目第二负责人长期从事计算流体动力学和气动声学的研究和实践，在重要国际刊物以及国际会议论文集上发表多篇论文，在发声模拟方面具备坚实基础和丰富的经验，指导本项目气体流场与声学模拟与分析。

5. 预初实验：

（1）课题组对 2 名上颌骨缺损患者使用鼻阻力计与声反射仪进行测试，结果（见

图 1）显示这类患者的鼻腔前部的容积，最狭窄处截面积和鼻阻力等，会因上颌骨的缺损及赝复体的初戴而发生改变。

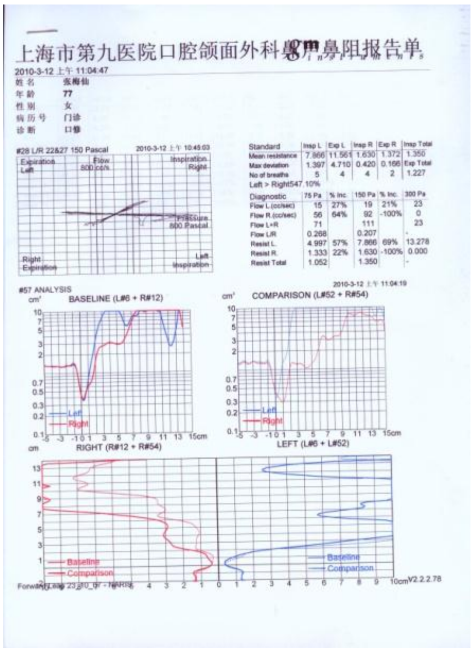

图 1. 患者鼻声鼻阻报告单

（2）课题预初对一例单侧上颌骨缺失患者的上呼吸道进行三维重建及数值模拟分析，获取患者上呼吸道气流流场分布模式（图 2，3）。

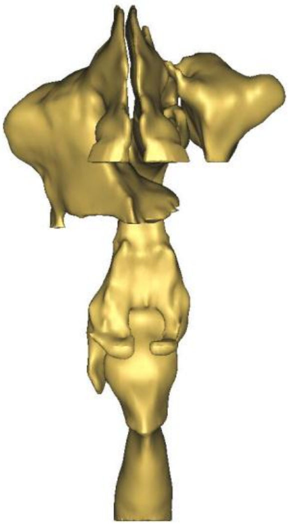

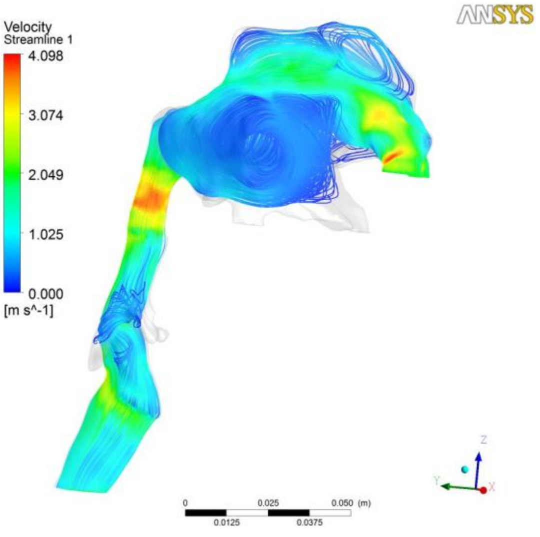

图 2.患者上呼吸道三维模型　　　图 3.气流流线图

（3）课题组通过图像处理软件，反求出可用于快速成型技术加工的上呼吸道空腔的三维模型。冠状位截面见图 4。

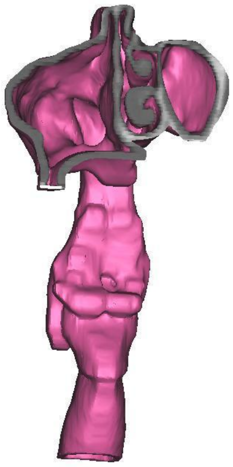

图 4.患者上呼吸道空腔三维模型

2、工作条件（包括已具备的实验条件，尚缺少的实验条件和拟解决的途径，包括利用国家实验室、国家重点实验室和部门开放实验室等研究基地的计划与落实情况）

已具备的实验条件

1. 课题在实验中需要一定量的志愿者和单侧上颌骨肿瘤全切除术患者，基于课题负责人在口腔肿瘤赝复领域积累的丰富的临床经验与良好的医患关系，基本可以得到建模的需要。

2.九院口腔修复科有颌面赝复数字化实验室，拥 Mimics 软件、快速成型机，医院拥有先进的鼻腔与呼吸功能测试仪。拥有自己的义齿加工实验室，能良好的完成阻塞器赝复体的制作。

3. 上海交通大学机械与动力工程学院在生物力学工程领域具有丰富的研究经验,在人体颅骨生物力学仿真、假体设计与生物力学评估等方面取得了重大的成果，对本研究阻塞器设计与建模有重要的指导作用；上海交通大学航空航天学院，在流体力学领域、声学领域有很强实力，参加多项 863 项目、国家自然基金项目，拥有 ACTRAN 等力学、声学分析软件，以及时间解析粒子图像速度仪、高频响动态压力传感器、声学麦克风等先进的硬件设施，为研究呼吸道流场、声场数据计算与分析提供实验条件。

尚缺少的实验条件

1. 图像处理软件、流体力学与声学软件需要进一步升级，以满足复杂管腔的建模、计算。同时对数值模拟得到的数据需要设备处理。

2. 用于流体仿真模型制作的快速成型材料。

3. 呼吸过程控制设备要自行开发制作。

拟解决的途径

1.对所需软件升级或与公司商定租用等。

2．购买适合制作实验流场的专用快速成型材料。

3．自行开发设计一系列光电学设备，形成有效的控制呼吸过程的实验流场仪器。

3、申请人简介（包括申请人的学历和研究工作简历，近期已发表与本项目有关的主要论著目录和获得学术奖励情况。论著目录要求详细列出所有作者、论著题目、期刊名或出版社名、年、卷（期）、起止页码等；奖励情况也须详细列出全部受奖人员、奖励名称等级、授奖年等）

项目负责人

焦婷：女，1972年出生，副教授，副主任医师，硕士生导师，中华口腔医学会口腔修复专业委员会青年委员，全国口腔职业医师考试专家组成员。1997年毕业于上海第二医科大学，获医学学士、口腔医学硕士学位。2003年毕业于上海第二医科大学，获口腔医学博士学位，师从张富强教授。2005年-2006年赴美国加州大学洛山矶分校（UCLA），师从国际颌面修复主席Dr Beumer教授，完成博士后、口腔颌面修复高级住院医师培训，并获得美国加州大学洛山矶分校住院医生资格证书。近3年内，在国内、外权威杂志上发表论文二十余篇，第一作者16篇，其中3篇为SCI收录，4篇为ISTP收录，负责和主要参加市科委、市教委及院级课题3项,2005年《颌面功能性赝复系统的开发和临床应用研究》 达到国际先进水平。同年荣获“上海市优秀青年医学人才培养计划”，2006年荣获上海市优秀研究生论文（博士），2007年获MIMICS Innovation Award，2009年荣获上海领军人才“后备队”专项基金。

研究方向

主攻口腔修复－颌面赝复学，从口腔修复的角度对肿瘤患者进行颌面赝复的序列治疗，对肿瘤患者术后美观、功能、语音、口腔微生态、及生物力学等方面进行研究，并对颌面赝复材料以及计算机辅助设计与制作在颌面赝复中的应用等进行研究。在国内率

先提出颌面缺损修复CAD/CAM系统，填补了我国颌面修复CAD/CAM技术的空白。

本项目中承担的任务

在本项目中承担课题总体设计、实施、指导，主要指导三维建模和气声场分析，设计阻塞器赝复体、指导临床操作。

近期已发表与本项目有关的主要论著

1. Jiao T, Chang TL, Caputo AA. Load transfer characteristics of unilateral distal extension removable partial dentures with polyacetal resin supporting components[J]. Aus Dent J 2009，54（2）：31-37(IF: 0.568)
2. Sun J, Jiao T, Tie Y, Wang DM . Three-dimensional finite element analysis of the application of attachment for obturator framework in unilateral maxillary defect[J].J Oral Rehabil.2008, 35(9):695-699. (IF: 1.200)
3. Jiao T, Zhang F, Huang X, Wang C. Design and Fabrication of auricular prostheses by CAD/CAM System[J]. Int J Prosthodont.2004,17(4):460-3.(SCI收录，IF：1.414)
4. 钱玉梅,陈丽萍,吴亚东,焦婷.人体上呼吸道三维数值模型的建立与气体流场数值模拟分析[J].上海口腔医学,2010,19(3):310-314.
5. 唐莹,焦婷.口腔鳞状细胞癌患者序列治疗中口腔生态系的改变[J] .国际口腔医学杂志,2009,36(4):416-419.
6. 焦婷,洪凌斐,孙健,张保卫,潘瑾,夏萌培.附着体修复单侧上颌骨缺损的光弹应力分析[J].上海交通大学学报(医学版),2008,28(11):1356-1359.
7. 焦婷,张富强. 应用核磁共振(MRI)采集及三维重建面部表面软组织形态的研究[J].口腔颌面修复学杂志,2007,8(4):265-267.
8. 孙健,焦婷,洪凌斐,铁瑛,王冬梅.单侧上颌骨缺损闭合式重建时赝复体的三维有限元分析[J].口腔颌面修复学杂志,2007,8(2):122-123.
9. 焦婷,孙健,洪凌斐,张富强,铁瑛,王冬梅.附着体应用于单侧上颌骨缺损修复的三维有限元分析[J].上海口腔医学,2006,15(5): 370-374.
10. 洪凌斐,孙健,焦婷,张保卫,铁瑛,王冬梅.单侧上颌骨缺损修复三维有限元模型的建立及力学分析[J].上海口腔医学,2006,15(4):403-406.
11. 孙健,姜卫东,焦婷,张富强,李静.附着体应用于单侧上颌骨缺损修复的模型设计与制作[J].口腔颌面修复学杂志, 2006,7(4): 258-260.

12. 焦婷,张富强,孙健.应用三维激光扫描仪采集及重建头面部软组织的研究[J]. 上海口腔医学, 2005,4(5):463-465.
13. 邢国芳,焦婷,孙健,蒋永林.单侧上颌骨切除术后赝复体修复患者的语音评价[J].上海口腔医学, 2005,14(4):352-354.
14. 焦婷,张富强,孙健. 计算机辅助设计领面缺损修复技术的开发应用研究[J].中华口腔医学杂志 2004, 39(2):257-259.

项目第二负责人

吴亚东: 上海交通大学航空航天学院博士后，2009年2月毕业于上海交通大学叶轮机械研究所，获工学博士学位，主要从事非定常流场和气动声学的研究，在重要国际刊物以及国际会议论文集上发表多篇论文，多篇被SCI和EI收录，申请专利多项。

本项目中承担的任务

在本项目中负责单侧上颌骨肿瘤患者阻塞器赝复体修复前后的上呼吸道气体流场与声场数值模拟分析指导。

近期发表与本项目有关的主要论著

1. Yadong WU, Xiaocheng ZHU, and Zhaohui DU. Experiment Investigation on Stator-rotor Interaction with Trailing Edge Blowing. International Conference on Power Engineering. Hangzhou, China, 2007, 5, 324-330. (EI: 20094412411809 SCI: BHP42)
2. Yadong Wu, Xiaocheng Zhu, and Zhaohui Du. Numerical investigation on the interaction of momentumless wake between rotor. Journal of Aerospace Engineering. (Revised)
3. Yadong WU, Guangyuan JIN, Hua OUYANG, Zhaohui DU. Experimental Investigations on Tip Leakage Flow and Noise in Skewed Blades. AIAA paper, 16th AIAA/CEAS Aeroacoustics Conference, AIAA 2010-3908. (EI: 20104913451373)
4. Yadong WU, Hua OUYANG, Jie TIAN, Zhaohui DU. PIV investigation of the flow field in a centrifugal impeller with unequally spaced blades, Journal of Power and Energy, 2008, 222:389-402.
5. Yadong WU, Xiaocheng ZHU, and Zhaohui DU，Experimental investigation on the momentumless wake using trailing edge blowing, Journal of Mechanical Engineering Science, 2008, 222:1477-1486.
6. Yadong WU, Xiaocheng ZHU, and Zhaohui DU, Experimental and Numerical

Investigation on Aeroacoustic Sound of Stator Wake/rotor Interaction with Trailing Edge Blowing, ASME paper, PROCEEDINGS OF THE ASME TURBO EXPO 2008, 6:779-787. (ISTP: BIT77)

7. Yadong WU, Xiaocheng ZHU, and Zhaohui DU, Experimental investigation on momentumless wake and its application in reduction of unsteady stator-rotor interaction, Proceeding of the Fifth International Conference on Fluid Mechanics, Shanghai, China, 2007, 5, 467-470.(ISTP:BHL40)
8. 吴亚东，田杰，竺晓程，杜朝辉. 带尾缘吹气的动静干涉流动的实验[J].航空动力学报,2011,(01):85-91.
9. 吴亚东,竺晓程,欧阳华,田杰,杜朝辉. 静子尾缘喷气后尾迹与动叶干涉噪声研究.工程热物理学报,2009,30(9):1482-1484.
10. 吴亚东,竺晓程,杜朝辉.基于粒子图像测速技术研究带尾缘吹气静子尾迹特征.推进技术,2008,29(2):230-234.
11. 吴亚东,竺晓程,杜朝辉.无动量损失尾迹的实验研究.实验流体力学,2007,22(3):30-34.

专利

1. 吴亚东,竺晓程,欧阳华,杜朝辉.带吸力边喷气的叶轮机械翼型.公开号 CN 101109395A，2008 年 1 月 23 日公开。
2. 竺晓程,吴亚东,杜朝辉,欧阳华. 带尾缘喷气叶轮机械翼型.公开号 CN 101109396A，2008 年 1 月 23 日公开。
3. 欧阳华,吴亚东,田 杰,杜 江,竺晓程 . 周向弯角可调式静叶,机构 申请号 200910049407.2，公开号 CN101539034。
4. 朱幼君,欧阳华,杜朝辉,竺晓程,吴亚东.扩散型消声器. 公开号：CN101418820，2009 年 4 月 29 日 公开。

项目第三负责人

熊耀阳：女，2000 年毕业于上海第二医科大学口腔医学专业，2008 年获博士学位，完成博士论文《结构光投影面部三维测量系统建立及应用研究》。研究领域：颌面赝复数字化信息采集、建模与图像处理。2006-2008 年，参与上海市信息化委员会专项资金项目《基于三维视觉测量的颜面缺损修复 CAD 系统的开发及其应用推广》，作为主要实验人员完成项目，并组织完成了项目的结题和验收工作。2009 年获国家自然基金青年基金

资助《基于多数字化信息采集的颌面部缺损赝复体系的研究》，项目批准号 30901694，主要承担课题设计、颌骨缺损信息采集、数字化图像融合技术实施以及临床测试工作。2009 年获 “中华口腔医学会——登士栢口腔医学青年人才奖”优胜奖。发表相关论文 5 篇，参编两部论著。

本项目中承担的任务

赝复体与声学模型图像处理与分析。

近期发表与本项目有关的主要论著

1. 熊耀阳,焦婷,张富强, 陈晓波,刁俊通. 快速成型技术在鼻赝复中的应用研究. 上海交通大学学报(医学版) , 2008,28(4):417-419.
2. 熊耀阳,焦婷,张富强. 结构光三维测量轮廓技术及快速成型技术在颌面赝复中的应用. 中国组织工程和临床康复, 2008,12(9):1705-1708.
3. 孙健,熊耀阳,张富强,陈晓波,刁俊通. 三维光学扫描及快速成型技术制作面部软组织三维树脂模型的初步研究.中华口腔医学杂志, 2007,42(7):403-405.
4. 熊耀阳,孙健,张富强,陈晓波,刁俊通. 应用结构光三维扫描技术重建面部软组织形态的初步研究.中华口腔医学杂志, 2007,42(6):340-342.
5. 熊耀阳,孙健,张富强.上海地区成人鼻面部的观察与测量.解剖学杂志, 2006; 29(6): 784-786.

主要参与者

陈晓波: 工科博士，现为上海交通大学机械与动力工程学院师资博士后。2001 年获上海交通大学机械工程学士学位，2002 年获上海交通大学工商管理学士学位。2003 年获美国密歇根大学机械工程硕士学位。2009 年获上海交通大学机械工程博士学位。主要研究方向为三维视觉测量。在该领域已刊登发表 SCI 收录论文 4 篇，累计他引 21 次；获得国家发明专利授权 4 项，专利公开 2 项，软件著作权 2 项。

本项目中承担的任务

体外实验流场分析、测量。

近期发表与本项目有关的主要论著

1. Chen Xiaobo, Xi Juntong, Ye Jin, et al., Accurate calibration for a camera-projector measurement system based on structured light projection, Optics and Lasers in Engineering,

2009, 47(3-4), 310-319. (ISI:000264096500005, EI: 090711904033).
2. Chen Xiaobo, Xi Juntong, Jiang Tao, et al., Research and development of an accurate 3D shape measurement system based on fringe projection: Model analysis and performance evaluation, Precision Engineering, 2008, 32(3), 215-221. (ISI: 000256518500007, EI: 081911243719).
3. Chen Xiaobo, Xi Juntong, Ye Jin, Phase error compensation method using smoothing spline approximation for a three-dimensional shape measurement system based on gray-code and phase-shift light projection, Optical Engineering, 2008, 47(11), 113601 (ISI:000261267800004).
4. Chen Xiaobo, Xi Juntong, Ye Jin, et al., Accuracy improvement for 3D shape measurement system based on gray-code and phase-shift structured light projection, in S. J. Maybank, M. Ding, F. Wahl and Y. Zhu, Proceedings of SPIE, V6788, MIPPR 2007, Wuhan, China, 2007, 67882C (ISI: 000252363600083, EI: 081811236233).
5. 胡民政，陈晓波，习俊通， 两轴转台结构光三维扫描仪及多视自动拼合，测试技术学报，2010，24(2)，161-169.

专利

1. 习俊通，陈晓波，熊耀阳等，用于患者颜面缺损修复的双单目白光三维测量系统，中国，发明专利，2008（授权号：ZL 200810038198.7）
2. 习俊通，陈晓波，熊耀阳等，测量颜面缺损患者面部三维形貌的方法，中国，发明专利，2008（授权号：ZL 200810038199.1）
3. 习俊通，陈晓波，熊耀阳等，双单目白光三维测量系统标定方法，中国，发明专利，2008（授权号：ZL 200810038222.7）

软件著作

1. 软件著作权登记：基于三维视觉测量的颜面缺损修复 CAD 系统 V1.0，2009（登记号：2009SR0764）
2. 软件著作权登记：基于轻量化模型的复杂曲面产品数字化检测系统[简称：SurfDIS]V1.0，登记号 2010SR073011）

4、**承担科研项目情况**（申请人正在承担或参加科研项目的情况，包括自然科学基金的项目。要注明项目的名称和编号、经费来源、起止年月、

与本项目的关系及负责的内容等）

项目负责人：焦婷

上海市教委创新课题重点项目（11ZZ102）"基于呼吸功能数值模拟的阻塞器优化设计与个性化制作"2011-2013年，第一负责人。主要承担课题总体设计、实施，阻塞器的优化设计和临床测试指导。

项目组主要参与人员：熊耀阳

国家自然基金青年基金(30901694) "基于多数字化信息采集的颌面部缺损赝复体系的研究"，2010-2012年，第一负责人，主要承担课题设计、颌骨缺损信息采集、数字化图像融合技术实施以及临床测试工作。

5、完成自然科学基金项目情况（对申请人负责的前一个已结题科学基金项目（项目名称及批准号）完成情况、后续研究进展及与本申请项目的关系加以详细说明。另附该已结题项目研究工作总结摘要（限 500 字）和相关成果的详细目录）

无

（三）经费申请说明（要求购置 5 万元以上固定资产及设备等，须逐项说明与项目研究的直接相关性及必要性）

经费按照《国家自然科学基金经费管理办法》填写，本课题未计划购置5万元以上固定资产及设备。

其他附件清单（附件材料复印后随纸质《申请书》一并上交）

（随纸质申请书一同报送的附件清单，如：具有中级技术职称申请者的推荐信或在职研究生申请项目的导师推荐信等。在导师的推荐信中，需要说明申请课题与学位论文的关系，承担课题后的工作时间和条件保证等。）

附件1：9th ISMR　论文录用与大会英语交流证书

附件2：6th IADR（中国分会）优秀青年学者奖证书

附件3：伦理委员会同意证明及受试者知情同意书

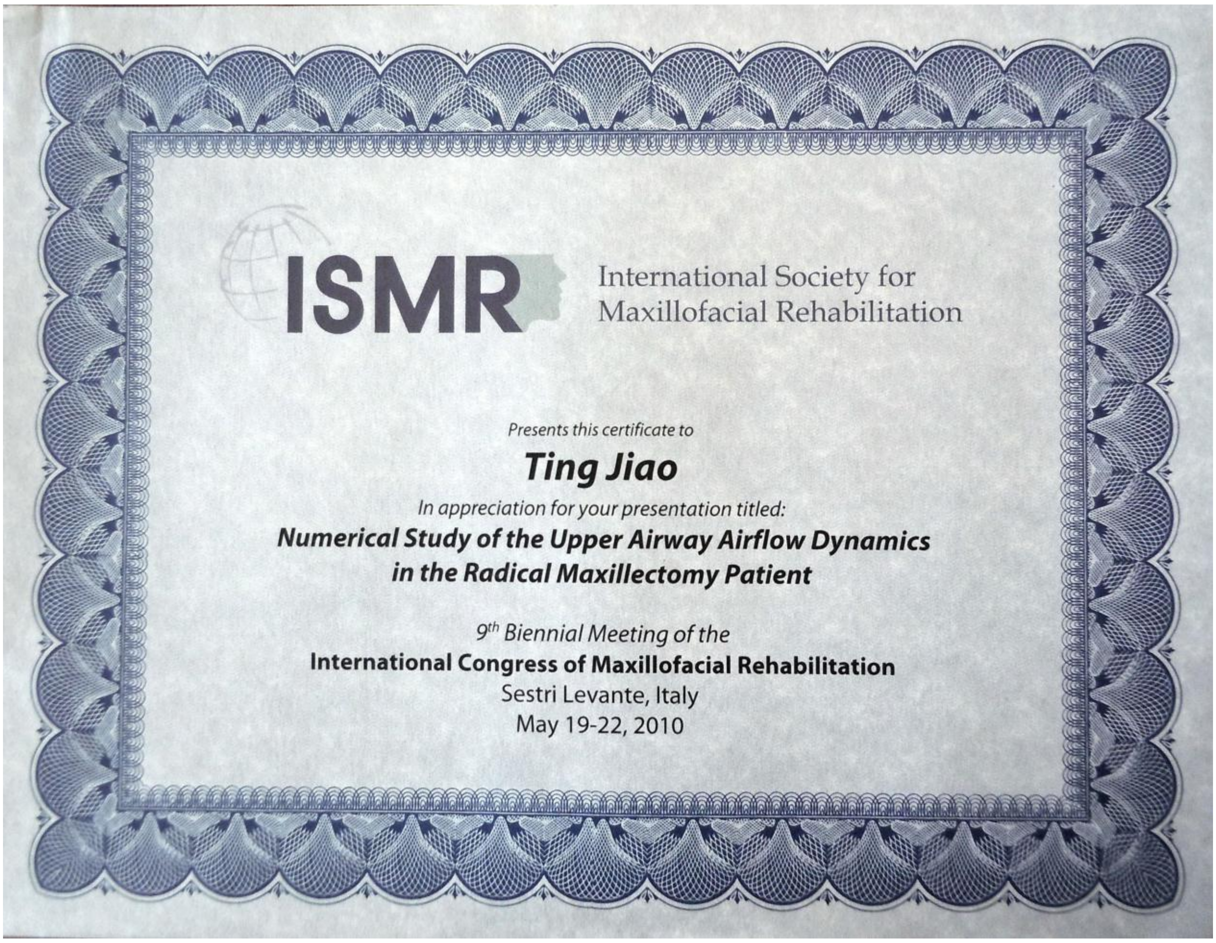

ISMR International Society for Maxillofacial Rehabilitation

Presents this certificate to

Ting Jiao

In appreciation for your presentation titled:

Numerical Study of the Upper Airway Airflow Dynamics in the Radical Maxillectomy Patient

9th Biennial Meeting of the

International Congress of Maxillofacial Rehabilitation

Sestri Levante, Italy

May 19-22, 2010

IADR CHINESE DIVISION
国际牙科研究协会中国分会

Certificate

This certificate is presented to Dr. Jiao Ting for participation in the 6th IADR Chinese Division Travel Award Competition, and obtained The Second-Class Prize.

President
IADR Chinese Division

Executive Director
IADR Chinese Division

2010-12-01
Xiamen, China

上海交通大学医学院附属第九人民医院伦理委员会科研伦理快速审查决定表

Scientific Research Projects Approval Determination of Independent Ethics Committee of Shanghai Ninth People's Hospital affiliated to Shanghai JiaoTong University, School of Medicine

沪九院科伦审[2011]8号（Number）

科研项目名称<br>**Project**	基于数值模拟的上颌骨缺损患者呼吸与发音变化机制研究<br>Numerical simulation on the respiration and speech mechanism compromised secondary to maxillectomy defect		
申请目的<br>**Project objective**	√项目申请 Project Application　□ 论文发表 Publications　□ 成果报奖 Outcome reward		
申请科室（专业）<br>**Division**	口腔修复	项目负责人<br>**Responsibility for project**	焦婷
伦理申请联系人<br>**Name of IEC Applicant**	焦婷	伦理申请联系人电话（手机）<br>**Tel / E-mail:**	13162548279
报送材料<br>**Data of material**	□ √科研项目立项标书（或申请书）及日期:<br>Establishment of project (Version Date)<br>□ √知情同意书及日期:<br>Informed consent (Version Date)<br>□ √参考文献或其他:<br>Reference and others		

快速审查意见（至少下列委员会授权快速审查人员中有 2 人对该项目进行审查）
Quick review reports (more than two committee member)

姓名<br>Name	职务<br>Position	工作部门/职称<br>Department/ Title	同意<br>Agreement	不同意<br>Disagreement	签名<br>Signatory
吴正一<br>Wu Zhengyi	委员<br>committee member	院长办公室/副研究员<br>Department of hospital general administration Office / Associate Professor	√		[signature]
陆尔奕<br>Lu Eryi	委员<br>committee membe	科研处/馆员<br>Department of research and education/ Associate Professor	√		[signature]
张少明<br>Zhang Shaoming	委员<br>committee membe	门诊办公室/副主任医师<br>Outpatient administration office/ Associate Professor			
阮 洪<br>Ruan Hong	委员<br>committee membe	护理部/副主任护师<br>Department of nursing/ Associate Professor of Nuring			
田卓平<br>Tian	委员<br>committee membe	医务处/教授<br>Department of medical			

1

Zhuoping		administration/ Professor			
伦理委员会意见<br>**Results of Independent Ethics Committee approval**	审查结果：<br>Result of IEC<br>（1）同意临床试验 ☑<br>Agreement to clinical trial<br>（2）作必要修改后同意 ☐<br>After review to do clinical tria<br>（3）不同意临床试验 ☐<br>No Agreement to clinical trial<br>（4）终止或暂停已批准的试验 ☐<br>Stop the clinical trial<br><br>九院伦理委员会（签章）<br>Shanghai Ninth People's Hospital，IEC<br>日期 Date: 2011.2.25.				
	修改意见：<br>Suggestion for revision:				

地址：上海市制造局路 639 号(200011)
Address: No. 639, Zhizaoju road, Shanghai, China，200011
电话：（021）23271699－5339
Tele: +86-021-23271699-5339
传真：（021）63136856
Fax: +86-021-63136856

（本决定将在九院医学伦理委员会审查会上通报，对不同意的项目，仍需要提交伦理审查委员会上审查；本决定复印件及相关资料由九院医学伦理委员会归档保存）

2

签字和盖章页(此页自动生成,打印后签字盖章)

检查保护

申 请 人：焦婷　　依托单位：上海交通大学
项目名称：基于数值模拟的上颌骨缺损患者呼吸与发音变化机制研究
资助类别：青年科学基金项目　　亚类说明：
附注说明：

申请人承诺：

我保证申请书内容的真实性。如果获得资助，我将履行项目负责人职责，严格遵守国家自然科学基金委员会的有关规定，切实保证研究工作时间，认真开展工作，按时报送有关材料。若填报失实和违反规定，本人将承担全部责任。

签字：

项目组主要成员承诺：

我保证有关申报内容的真实性。如果获得资助，我将严格遵守国家自然科学基金委员会的有关规定，切实保证研究工作时间，加强合作、信息资源共享，认真开展工作，及时向项目负责人报送有关材料。若个人信息失实、执行项目中违反规定，本人将承担相关责任。

编号	姓　名	工作单位名称	项目分工	每年工作时间(月)	签　字
1	吴亚东	上海交通大学	流声场分析指导	4	
2	熊耀阳	上海交通大学	赝复体与声学图像处理	4	
3	陈丽萍	上海交通大学	临床呼吸测试	4	
4	陈晓波	上海交通大学	体外实验流场测量分析	4	
5	钱玉梅	上海交通大学	三维建模与流场分析	6	
6	董晛	上海交通大学	实验流场中的呼吸模拟	8	
7	盖德倩	上海交通大学	三维建模与声学分析	8	
8	王昊	上海交通大学	流场与声场计算模拟	8	
9					

依托单位及合作研究单位承诺：

已按填报说明对申请人的资格和申请书内容进行了审核。申请项目如获资助，我单位保证对研究计划实施所需要的人力、物力和工作时间等条件给予保障，严格遵守国家自然科学基金委员会有关规定，督促项目负责人和项目组成员以及本单位项目管理部门按照国家自然科学基金委员会的规定及时报送有关材料。

依托单位公章　　合作研究单位公章 1　　合作研究单位公章 2

日期：　　日期：　　日期：
